# Glycosylation modulation as a therapeutic strategy for neuroinflammatory disorders: The potential of Tanshinone IIA

**DOI:** 10.1016/j.isci.2026.116012

**Published:** 2026-05-20

**Authors:** Yahui Li, Kunfeng Duan, Guangyuan Liu, Jingwen Yan, Qian Wang, Yuyu Zhang, Yuran Wang, Bowen Guo, Panpan Zhang, Wei Zhang, Dezhi Kong

**Affiliations:** 1 Department of Pharmacology of Chinese Materia Medica, Institution of Chinese Integrative Medicine, School of Chinese Integrative Medicine, Hebei Medical University, Shijiazhuang 050017, China; 2 Key Laboratory of Tranquilizing TCM, Hebei Provincial Administration of Traditional Chinese Medicine, Shijiazhuang 050017, China; 3 Pharmacy Department, Hebei Key Laboratory of Stomatology, Hebei Clinical Research Center for Oral Diseases, School and Hospital of Stomatology, Hebei Medical University, Shijiazhuang 050000, China; 4 Pharmacy Department, The Third Hospital of Hebei Medical University, Shijiazhuang 050051, China; 5 Key Laboratory of Neural and Vascular Biology, Ministry of Education of China, Shijiazhuang, China; 6 School of Traditional Chinese Medicine, Hebei University of Chinese Medicine, Shijiazhuang 050200, China

**Keywords:** therapy, protein, molecular interaction

## Abstract

Neuroinflammation is a pivotal pathological process underlying numerous neurological disorders. While the interplay between neuroinflammation and glycosylation is gaining increasing attention, it remains unclear whether modulating glycosylation can effectively inhibit neuroinflammation. To address this, our study employed a combined proteomic and glycoproteomic approach to investigate the role of protein glycosylation in lipopolysaccharide (LPS)-induced neuroinflammation models. There was a significant association between the changes we observed and pathways related to glycation and neuroinflammation. Moreover, during neuroinflammation, protein glycosylation levels exhibit fluctuations. Crucially, we demonstrated that the pan-glycosylation inhibitor NGI-1 effectively suppressed the inflammatory response. We subsequently screened Tanshinone IIA (STS), identifying it as a potent therapeutic candidate for neuroinflammatory disorders. Tanshinone IIA exerts its anti-inflammatory effects partially through the targeted inhibition of Hsp90b1 glycosylation at position 217, thereby attenuating the activation of the NF-κB pathway. Collectively, our findings offer novel insights into the therapeutic potential of targeting glycosylation modifications in neuroinflammation.

## Highlights

• Multiomics analysis reveals dynamic changes in glycosylation during neuroinflammation
• Tanshinone ⅡA inhibits neuroinflammation by modulating protein glycosylation
• Inhibiting glycosylation at position 217 of Hsp90b1 alleviates neuroinflammation

## Graphical abstract

**Figure:**
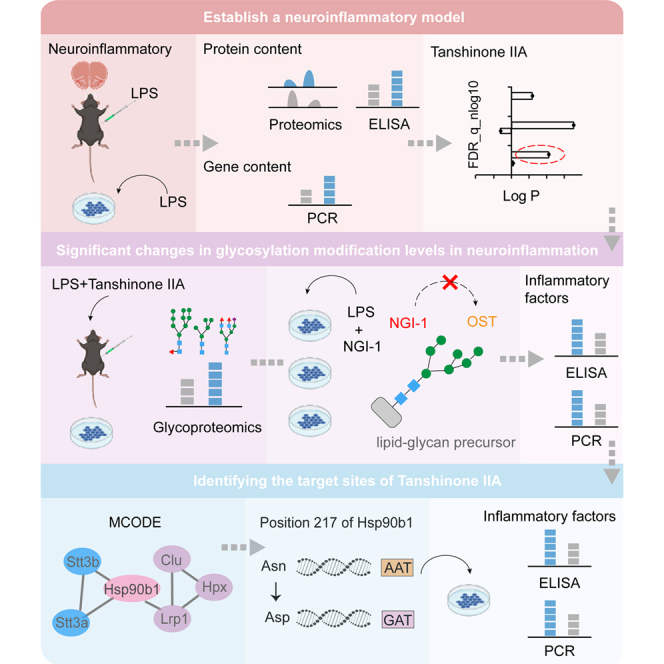

## Introduction

Neuroinflammation, characterized by the activation of immune cells within the central nervous system (CNS), plays a dual role in various neurological disorders, capable of both protecting the nervous system and promoting disease progression.^1^ In its acute phase, neuroinflammation is generally considered a crucial process for host defense and tissue repair. However, when this inflammatory response becomes dysregulated, excessive, or chronically sustained, it transitions from being protective to destructive, leading to neuronal damage, synaptic dysfunction, and neurodegeneration.^2^^,^^3^ Accumulating evidence suggests that chronic neuroinflammation is a common underlying driver of numerous neurodegenerative diseases.^4^^,^^5^^,^^6^ Despite the widely recognized pivotal role of neuroinflammation in neurological diseases, safe and effective therapeutic agents remain largely elusive in clinical practice. Furthermore, the long-term management of chronic neuroinflammation is complicated by the systemic side effects of existing anti-inflammatory drugs, precluding their widespread or prolonged use.^7^ Therefore, a deeper understanding of the molecular mechanisms underlying neuroinflammation and the development of targeted therapeutic strategies represents significant challenges in the field of neuroscience.

Significant advancements have been made in recent years in unraveling the mechanisms of neuroinflammation. Central to this understanding are the NF-κB pathway, mitogen-activated protein kinase (MAPK) pathway, and NOD-like receptor protein 3 (NLRP3) inflammasome, which serve as core regulatory elements.^8^^,^^9^ Moreover, specific proteins such as amyloid-beta (Aβ), alpha-synuclein (α-synuclein), and HMGB1 contribute to neuroinflammatory disease progression by activating Caspase-1, which, in turn, induces the maturation and release of pro-inflammatory cytokines interleukin-1β (IL-1β) and interleukin-18 (IL-18).^10^^,^^11^ In addition to these defined molecular pathways, broader cellular and systemic factors, including mitochondrial dysfunction, dysregulated lysosomal autophagy, endoplasmic reticulum (ER) stress, and interactions within the gut microbiota-brain axis, have also been demonstrably linked to the initiation and perpetuation of neuroinflammation.^12^ However, further investigation is needed to determine whether these pathways or targets can serve as viable drug targets.

In recent years, the intricate relationship between neuroinflammation and glycosylation has garnered increasing attention. Aberrant glycosylation is increasingly recognized as playing a critical role in both neuroinflammation and the progression of neurodegenerative diseases.^13^ Abnormal protein glycosylation is a key feature of Alzheimer’s disease (AD) pathogenesis. Both glycomic and glycoproteomic analyses have revealed widespread alterations in overall glycan profiles and specific glycosylation sites within AD brains.^14^ N-glycosylated Tau is detected in AD brain tissue but is absent in healthy counterparts.^15^ Among various glycosylation types, O-linked N-acetylglucosamine (O-GlcNAc) modification, a dynamic and reversible post-translational modification, has been shown to play a pivotal role in cellular stress responses, signal transduction, and inflammation regulation.^16^ Changes in O-GlcNAc modification levels can significantly impact the activity of inflammation-related pathways such as NF-κB and MAPK, consequently modulating microglial activation and the secretion of pro-inflammatory cytokines.^17^ Beyond O-GlcNAc, N-glycosylation also occupies a crucial position in neuroinflammation. Studies have revealed a significant reduction in the abundance of sialylated and core fucosylated N-glycan structures in neuroinflammatory regions of rat brain tissue.^18^ Furthermore, other glycosylation patterns, including sialylation, have been demonstrated to regulate neuronal survival, synaptic plasticity, and the progression of neuroinflammation.^19^^,^^20^ Intriguingly, glycosylation appears to exert a dual role in neuroinflammation. On one hand, it can exacerbate the over-activation of pro-inflammatory factors, leading to neuronal damage.^21^ On the other hand, it influences cell adhesion and migration, thereby modulating neuro-repair processes.^22^ While these findings provide significant insights into the pathophysiological roles of glycosylation in neuroinflammation, the efficacy of specific inhibition or modulation of particular glycosylation modifications in suppressing neuroinflammation and improving neurological function, as well as their underlying molecular mechanisms, remains to be thoroughly investigated.

Given the intricate mechanisms of neuroinflammation and the limitations of current therapeutic agents, exploring natural products for the treatment of neuroinflammation represents a promising research avenue.^23^ In contrast to synthetic compounds, many natural products and their derivatives, exemplified by polyphenolic compounds such as curcumin,^24^ resveratrol,^25^ and quercetin.^26^ possess distinctive bioactivities and favorable safety profiles, making them particularly suitable for managing chronic neuroinflammatory conditions. This study aims to elucidate the precise role of aberrant glycosylation in neuroinflammation and, based on protein expression changes in lipopolysaccharide (LPS)-induced models, to identify and mechanistically validate natural compounds effective in ameliorating neuroinflammation via glycosylation modulation. Ultimately, this research seeks to provide novel insights and therapeutic strategies for neuroinflammation-related diseases.

## Results

### LPS-induced proteomics uncover potential links between neuroinflammation and glycosylation

**Figure 1:**
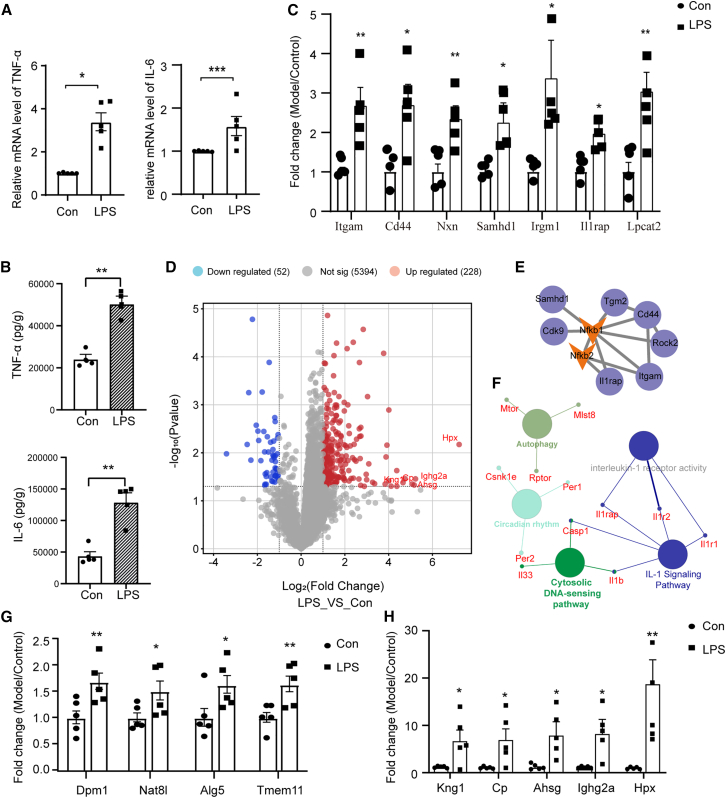
LPS-induced neuroinflammation in C57BL/6J mice and the potential links between neuroinflammation and glycosylation uncovered by proteomics (A) Effects of LPS infection on mRNA levels of cytokines TNF-α and IL-6 in Con and LPS group. Each group contained 5 samples. Four replicate wells were set up per sample. (B) ELISA results showed the protein expressions of TNF-α and IL-6. (C) Protein expression levels of Itgam, CD44, Nxn, Samhd1, Irgm1, IL1R1, and Lpcat2 changed in each group. (D) Differential gene expression distribution between Con and LPS groups. Each gene is represented by a dot. The top 5 significant upward adjustments were all for glycoproteins: Hpx, Ighg2a, Ahsg, Cp, and Kng1. The upregulated genes are indicated by red dots, the downregulated genes are represented by blue dots, and non-differential genes are indicated by gray dots. (E) NF-κB related PPI networks constructed. Each node represents a protein. (F) The enrichment pathways based on different proteins were generated using ClueGO in cytoscape. Node colors represent the pathway terms, and the size of the nodes indicates the significance of the term (larger means more significant). (G) Glycosylation-associated protein expression levels of Dpm1, Nat8l, Alg5, and Tmem11 changed in each group. (H) Glycoprotein expression levels of Kng1, Cp, Ahsg, Ighg2a, and Hpx changed in each group. Each group contained 5 samples. Error bars represent mean ± standard error (SE). Statistical analysis was performed by *t* test. *p* values indicating significance are depicted as follows: ∗*p* < 0.05, ∗∗*p* < 0.01, and ∗∗∗*p* < 0.001, ns is non-significant vs*.* LPS group.

To ensure the successful preparation of neuroinflammatory model, we quantified the mRNA and protein expression levels of pro-inflammatory cytokines in mice cerebral cortex by qRCR and ELISA assay. The mRNA and protein levels of IL-6 and TNF-α were significantly increased by LPS treatment (Figures 1A and 1B). The results indicate that LPS can induce neuroinflammation in mice, consistent with previous studies.^27^^,^^28^

Our proteomic analysis quantified 6,426 proteins. Both partial least squares-discriminant analysis (PLS-DA) and heatmap analysis revealed significant alterations in the proteome of the neuroinflammation group compared to the vehicle group, with high intra-group reproducibility (Figures S1A and S1B).

Compared with the vehicle group, the expression of 181 proteins was significantly changed in the neuroinflammation model group (with a fold change >2 and a threshold of *p* < 0.05) with 95 up-regulated and 86 down-regulated proteins. We found that the inflammation-related proteins were dysregulated in model mice (Figures 1C and 1D), and were closely related to Nfkb (Figure 1E). Itgam (Figure 1C) was significantly up-regulated in the neuroinflammation model group. Cd44 (Figure 1C), which is associated with the inflammatory response, was also significantly up-regulated in the neuroinflammation model group.^29^ Nxn (Figure 1C) functions as a redox-dependent negative regulator of the Wnt signaling pathway, interfering with the course of the inflammatory response through the Wnt signaling pathway. Samhd1 (Figure 1C) inhibits TAK1 activation and TRAF6 signaling in response to pro-inflammatory stimuli, and through a negative regulatory mechanism, Samhd1 is involved in inflammation, thereby maintaining body homeostasis.^30^ Irgm1 (Figure 1C) expression is increased in inflammation by promoting M1 macrophage activation and thus, production of pro-inflammatory cytokines.^31^ Il1rap binds to IL-1β to form a high-affinity interleukin-1 receptor complex, which mediates interleukin-1-dependent activation of NF-κB and other pathways. Lpcat2 (Figure 1C) exhibits acyltransferase and acetyltransferase activities, which are enhanced in response to inflammatory stimuli and promote the release of the pro-inflammatory lipid mediator platelet-activating factor (PAF).^32^ The abnormally expressed proteins put the organism in a neuroinflammatory activation phase by regulating a variety of mechanisms.

Volcano plots were drawn to show differentially expressed proteins in the brains of LPS-treated mice. Protein-protein interaction (PPI) network analysis for the differential expressed proteins revealed that IL-1 signaling pathway, cytosolic DNA-sensing pathway, and autophagy pathway mainly participated in LPS-induced neuroinflammation (Figure 1F). Proteins Hpx, Ighg2a, Ahsg, Cp, and Kng1 that were significantly up-regulated more than 6-fold compared to controls were glycosylated modified proteins (Figures 1D and 1H).

Interestingly, upon analyzing the proteins in Figures 1E–1H using UniProt and NetNGlyc-1.0, we discovered that these proteins are predominantly glycosylated. In addition, we found that protein expression levels associated with the glycosylation process were significantly up-regulated in the model group (Figure 1G). Dpm1 is a mannosyl donor in the pathway leading to protein N-glycosylation, glycosylphosphatidylinositol membrane anchoring, and O-mannosylation. Nat8l catalyzes the synthesis of NAA (N-acetylaspartate acid) from L-aspartate and acetyl-CoA.^33^ Alg5 is dolichyl-phosphate beta-glucosyltransferase that operates in the biosynthetic pathway of dolichol-linked oligosaccharides, the glycan precursors employed in protein asparagine (N)-glycosylation. Tmem115 may indirectly play a role in protein glycosylation in the Golgi. Therefore, these results indicate a potential link between neuroinflammation and glycosylation modifications, underscoring the necessity for comprehensive glycoproteomic studies in neuroinflammation models.

### Glycoproteomics of cerebral cortex in LPS-induced neuroinflammatory mice

**Figure 2:**
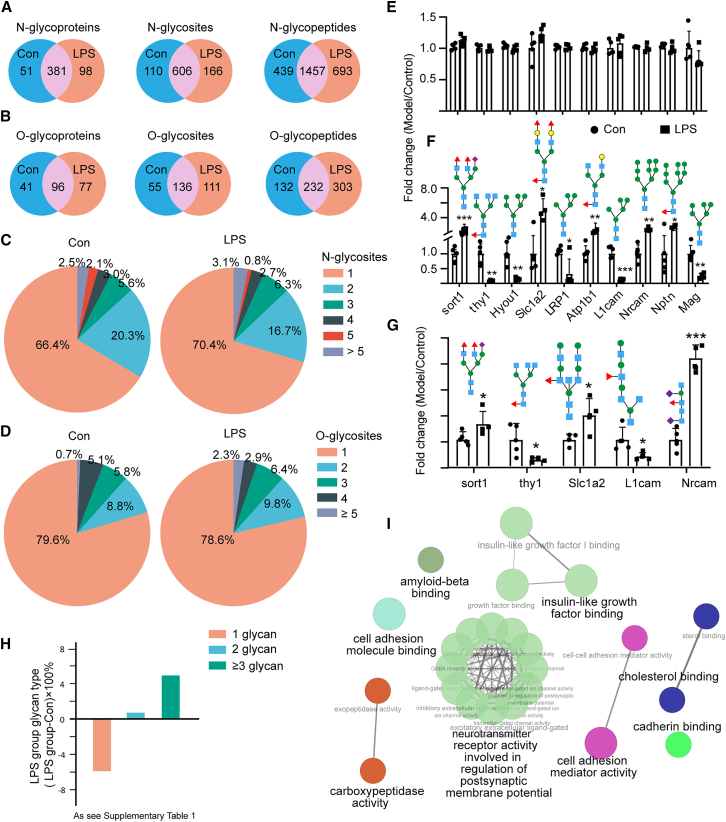
Qualitative and quantitative glycoproteomics analysis of LPS-induced neuroinflammation in mice (A) Comparisons of identified, N-glycoproteins, N-glycosites, and N-glycopeptides in LPS vs in control brains. (B) Comparisons of identified O-glycoproteins, O-glycosites, and O-glycopeptides in LPS vs*.* in control brains. (C and D) Pie charts showing the proportions of singly and multiply N-glycosylated (C) and O-glycosylated (D) proteins. (E–G) Detection of protein levels (E), N-glycan levels (F), and O-glycan (G) levels using LC-MS method. (H) Percentage of glycan content attached to glycosylation sites; vertical coordinate is (LPS group - control group) × 100%. (I) The enrichment pathways based on different glycoproteins were generated using ClueGO in cytoscape. Node colors represent the pathway terms and the size of the nodes indicates the significance of the term (larger means more significant). The glycan symbols are as follows: green circle for Hex, yellow circles for pentoses, blue square for HexNAc, purple diamond for sialic acids, and red triangle for fucose. Each group contained 5 samples. Error bars represent mean ± standard error (SE). Statistical analysis was performed by *t* test. *p* values indicating significance are depicted as follows: ∗*p* < 0.05, ∗∗*p* < 0.01, and ∗∗∗*p* < 0.001 vs*.* LPS group.

The objective of this study was to characterize the mouse cerebral cortex glycoproteome and identify protein glycosylation aberrations and their affected biological processes in neuroinflammation. We used our previous ZIC-HILIC-HCD-Orbitrap method to perform unbiased, large-scale, N-glycoproteome profiling analysis of mouse cerebral cortex samples from neuroinflammation and control cases.^34^ We identified 2,589 N-glycopeptides (hereafter referred to as N-glycopeptides); 530 N-glycoproteins; and 882 N-glycosylation sites (Figure 2A). We also identified 667 O-glycopeptides (hereafter referred to as O-glycopeptides), 214 O-glycoproteins, and 302 O-glycosylation sites (Figure 2B). In the control group, 66.4% of N-glycoproteins had 1 N-glycosylation site and 2.5% had more than 5 N-glycosylation sites; and 79.6% of O-glycoproteins had 1 O-glycosylation site, and 0.7% of O-glycosylated proteins had more than 5 O-glycosylation sites (Figures 2C and 2D). In the neuroinflammation model group, 70.4% of N-glycoproteins had 1 N-glycosylation site and 3.1% had more than 5 N-glycosylation sites; 78.6% of O-glycoproteins had 1 O-glycosylation site and 2.3% had more than 5 O-glycosylation sites (Figures 2C and 2D). The results indicated that protein glycosylation modifications were altered in the brain tissue of LPS-induced inflammation mice, including alterations in the number of glycoproteins, glycopeptides, and glycosylation sites.

In the glycoprotein enrichment results, the expression of the glycan content of Itgam was up-regulated, consistent with the changes in the whole protein (Figure S1C). We also found that some of the proteins that were not differentially expressed in the model group vs. the control group were also associated with inflammation (Figure 2E). The changes in the content of these proteins were not statistically significant between the normal and model groups, but the glycan content was significantly altered. Among them, the N-glycan expression of sort1, Slc1a2, Atp1b1, Nrcam, and Nptn was up-regulated and that of thy1, Hyou1, Lrp1, L1cam, and Mag was down-regulated in the LPS neuroinflammatory group compared with the control group (Figure 2F). The O-glycan expression of sort1, Slc1a2, and Nrcam was up-regulated and that of thy1 and L1cam was down-regulated in the LPS neuroinflammatory group compared with the vehicle group (Figure 2G). It is interesting to note that these glycosylated modified proteins play important roles in both the immune system and inflammation.^35^^,^^36^^,^^37^ Thus, glycosylation modifications may play a crucial role in inflammation by modulating glycan levels.

Glycans are recognized by glycan-binding proteins, such as the selectins, to induce inflammation.^38^ The ratio of ≥2 glycans were elevated in the neuroinflammation model group compared to the normal group (Figure 2H; Table S1). This is not a change in protein quantity, but rather an increase in glycans at the same glycosylation site, i.e., an increase in glycan occupancy. This finding provides powerful evidence that glycosylation induces neurological damage, and the results indicate that glycosylation modification levels are activated during neuroinflammation.

Compared with the control group, the expression of glycans content on 208 glycoproteins was significantly changed (with a fold change >2 and a threshold of *p* < 0.05) in the neuroinflammation model group. These glycoproteins, whose polysaccharide content undergoes significant changes, participate in multiple biological functions (Figure 2I). The presence of amyloid beta binding in the functional enrichment results reinforces the involvement of glycosylation modifications in neuroinflammation.

### LPS-induced neuroinflammatory model in BV2 cells

**Figure 3:**
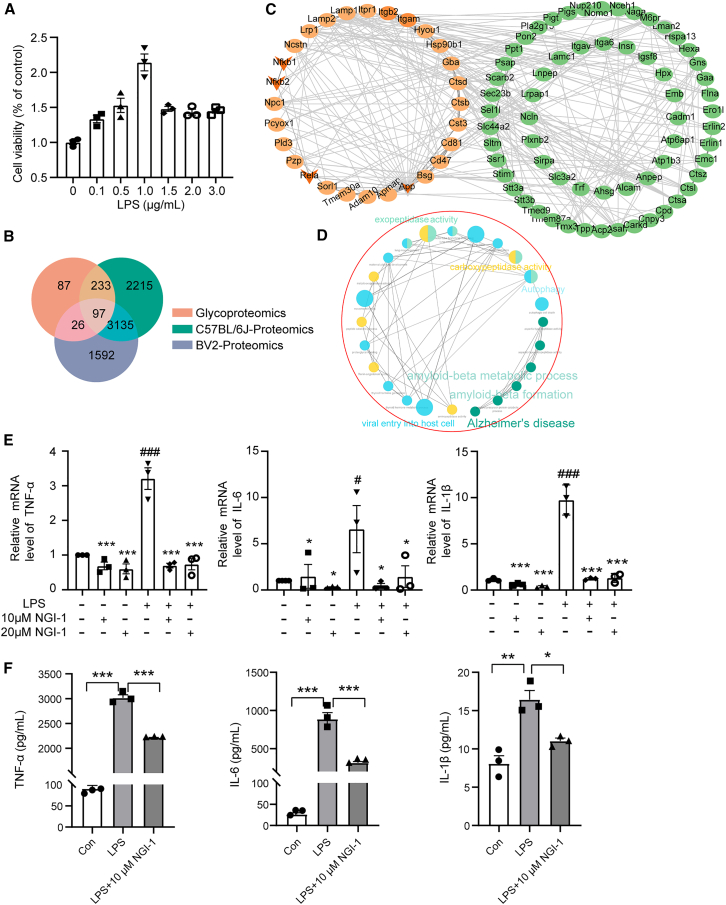
The LPS-induced neuroinflammation in BV2 cells was inhibited by NGI-1 (A) Effect of a series of doses (0.1, 0.5, 1.0, 1.5, 2.0, and 3.0 μg/mL) of LPS on cell viability for 24 h. (B) The overlap of proteins in the concentration of mice proteomic data (green), glycoproteomics data (red), and BV2 cell proteomics data (purple). (C) NF-κB related PPI networks constructed. Each node represents a protein. The legend explains the meanings of different connections between nodes. (D) The enrichment pathways based on co-glycoproteins were generated using ClueGO in cytoscape. (E) Effects of NGI-1 on mRNA levels of cytokines TNF-α, IL-6, and IL-1β in Con and LPS group. (F) ELISA results showed the level of TNF-α, IL-6, and IL-1β in the supernatant of BV2 microglias. Each group contained 3 samples. Error bars represent mean ± standard error (SE). Statistical analysis was performed by one-way ANOVA. *p* values indicating significance are depicted as follows: ^#^*p* < 0.05, ^##^*p* < 0.01, and ^###^*p* < 0.001 vs*.* control; ∗*p* < 0.05, ∗∗*p* < 0.01, and ∗∗∗*p* < 0.001 vs*.* LPS group.

BV2 cells are widely used to study the role of microglia in neuroinflammatory processes, such as those seen in neurodegenerative diseases, brain injury, and neurological disorders. Firstly, to determine the effect of LPS on cell viability, BV2 cells were treated with 0 μg/mL (as a vehicle), 0.1, 0.5, 1.0, 1.5, 2.0, and 3.0 μg/mL LPS for 24 h, and analyzed using the CCK-8 assay. The results showed that LPS promoted the proliferation of BV2 cells (Figure 3A). This was an interesting phenomenon showing LPS induced activation of BV2 cell proliferation.^39^ We chose the LPS dose of 1 μg/mL, which is reported as commonly used in the literature.^40^

Then, we performed proteomic analysis of BV2 cells to identify protein aberrations and their affected biological processes in the BV2 neuroinflammation model. PLS-DA and hierarchical cluster analysis (HCA) were used to identify the vehicle group and neuroinflammation model group (Figures S1D and S1E). These results indicate that protein levels were significantly altered in the neuroinflammation model group compared to the vehicle group, and the samples within each group were well parallelized. Proteomic analysis revealed that LPS treatment of BV2 cells induced a neuroinflammatory response, characterized by immune system disruption and dysregulated cytokine release. Ddx58 acts as an innate immune receptor and induces the production of type I interferon and pro-inflammatory cytokines,^41^ and Ddx58 levels were significantly increased in the BV2 neuroinflammation model group compared to the vehicle group, indicating that LPS promotes inflammatory progression. It has been reported that Sqstm1 binds to the membrane receptor INSR to activate glycolysis, leading to the production of NF-κB dependent pro-inflammatory cytokines,^42^ and elevated levels of Sqstm1 suggest that LPS contributes to neuroinflammatory responses through the NF-κB pathway. The signaling pathways of NF-κB include classical and nonclassical pathways, and among the nonclassical pathways, Nfkb2 is a core activator of genes involved in inflammation and immune function.^43^ In the model group, Nfkb2 was significantly up-regulated, indicating that the construction of the neuroinflammation model was successful. In addition, Fyb1, Pik3ap1, Tnip1, CD14, Slpi, and Tlr2 were all up-regulated in the neuroinflammatory response, consistent with the reported results^44^^,^^45^^,^^46^ (Figure S1F). It was shown by multiple sources of data that a successful model of neuroinflammation in BV2 cells has been established.

A cross-comparison between the proteome of the *in vitro* BV2 model and the proteome/glycoproteome of the *in vivo* mouse neuroinflammation model identified an overlap of 97 glycoproteins (Figure 3B). Functional analysis of these 97 glycoproteins revealed significant enrichment in positive regulation of leukocyte mediated cytotoxicity, AD, amyloid-beta formation, and amyloid-beta metabolic process (Figure 3C). This is consistent with experimental results in animals. Multiple pieces of evidence suggest that neuroinflammation is closely related to AD.^47^^,^^48^^,^^49^^,^^50^ By using STRING network analysis to observe the relationship between 97 shared glycoproteins with Rela, Nfkb1, Nfkb2 (primary regulatory genes in the NF-κB pathway), and App (a primary regulatory gene in the AD), we found that about 50% of the glycoproteins were tightly associated with Nfkb1, Nfkb2, and App (Figure 3D), suggesting that enriched glycoproteins play an important role in neuroinflammation.

### Glycosylation inhibitors can inhibit the production of inflammatory factors

To further verify the relationship between neuroinflammation and protein glycosylation modification, we added NGI-1 into BV2 cells. NGI-1 is a potent inhibitor of oligosaccharyltransferase (OST) that directly targets and blocks the function of the OST catalytic subunits STT3A and STT3B. By inhibiting OST, it affects the N-glycosylation process. Based on the effects of different concentrations of NGI-1 on cell viability, we found that the inhibitory effect of NGI-1 on cell viability was dose dependent, as shown in Figure S1G. We chose the concentrations of 10 and 20 μM NGI-1 (half inhibitory concentration) for the subsequent experiments.^51^ BV2 cells were pretreated with NGI-1 for 2 h before being exposed to LPS for 24 h. Following agglutinin-based enrichment of glycoproteins, we found that NGI-1 treatment inhibited protein glycosylation, leading to a decreased abundance of glycoproteins (Figure S1H). Importantly, this effect was independent of any general suppression of global protein synthesis by NGI-1 (Figures S1I–S1K). The results showed that LPS obviously promoted BV2 microglial activation by upregulating inflammatory mediators including TNF-α, IL-6, and IL-1β, whereas pretreatment with NGI-1 significantly decreased TNF-α, IL-6, and IL-1β secretion compared with the LPS-treated group (Figures 3E and 3F). Meanwhile, NGI-1 also significantly decreased the mRNA levels of TNF-α, IL-6, and IL-1β compared with the vehicle group (without LPS treatment, Figure 3E). This experimental result further confirms that effective treatment of neuroinflammation can be achieved by modulating the level of protein glycosylation modifications.

### Tanshinone IIA: An effective drug for neuroinflammatory disorders based on neuroinflammatory proteomics

**Figure 4:**
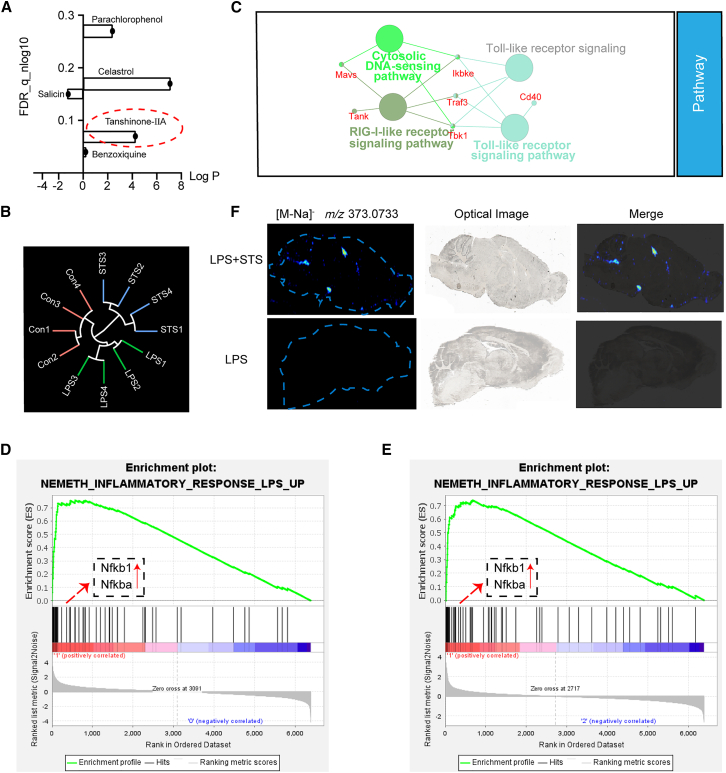
Tanshinone IIA attenuates neuroinflammation (A) Connectivity map (CMap) results, the *x* axis is the lipid-water distribution coefficient (logP), the *y* axis is false discovery rate (FDR). (B) The 12 samples were analyzed by unsupervised hierarchical cluster analysis. Each group contained 4 samples. (C) The enrichment pathways based on different proteins (STS group vs*.* LPS group) were generated using ClueGO in Cytoscape. (D and E) GSEA was applied for functional annotation (left: Con vs*.* LPS; right: STS vs*.* LPS). (F) Distribution of Tanshinone ⅡA in brain tissue.

Based on the results from neuroinflammatory proteomics, we screened the active traditional Chinese medicine (TCM) ingredients through Connectivity Map tools. Considering the reliability of the results and the logP values of the ingredients, Tanshinone IIA was identified as the best candidate (Figure 4A).

We examined the therapeutic effect of Tanshinone IIA on LPS-induced neuroinflammation mice; the levels of TNF-α, IL-6, and IL-1β in the cerebral cortex were significantly decreased compared with the LPS-induced model group (Figures S2A and S2B). The results of the experiments show that the ability of Tanshinone IIA to improve neuroinflammation is indisputable.

The protein alteration profile was investigated in the LPS-induced model groups treated with or without Tanshinone IIA (Figure 4B). The significantly changed proteins were subjected to Gene Ontology (GO) and Kyoto Encyclopedia of Genes and Genomes (KEGG) enrichment analyses and visualized by Cytoscape (Figure 4C). It was demonstrated that LPS could successfully induce neuroinflammation and that Tanshinone IIA could reduce LPS-induced neuroinflammation.

Tanshinone IIA (200 μM or 300 μM) mitigated LPS-induced neuroinflammation in BV2 cells (Figures S2C–S2E). This anti-inflammatory effect was confirmed in primary microglia, where Tanshinone IIA also attenuated the production of pro-inflammatory cytokines (Figure S4C). Then, we perform the proteomic study in the vehicle group, the neuroinflammation model group, and the Tanshinone IIA group. PLS-DA and heatmap analysis were used to distinguish the three groups (Figures S3A and S3B). The 7,189 proteins identified in three groups were analyzed using gene set enrichment analysis (GSEA). The inflammation-associated pathways were significantly enriched (Figures 4D and 4E). The differentially expressed proteins (with a fold change >2 and a threshold of *p* < 0.05) were analyzed for GO enrichment using the Microbiotics platform. This analysis was performed for the control vs. neuroinflammation group, as well as the Tanshinone IIA-treated group vs. the neuroinflammation group, as shown in Figures S3C and S3D. In KEGG-enriched pathways (treatment group vs. model group), it was notable that various types of N-glycans biosynthesis pathways (*p* = 0.054) were enriched (Table 1), suggesting that both LPS-induced neuroinflammation as well as Tanshinone IIA treatment of neuroinflammation may affect protein glycosylation modifications.

**Table 1:** The list of KEGG enrichment pathways

Description	Gene Ratio	Bg Ratio	*p* value
Coronavirus disease - COVID-19	13/85	248/9040	4.64281E−07
Measles	10/85	146/9040	9.86007E−07
Hepatitis C	10/85	165/9040	3.00911E−06
Influenza A	10/85	173/9040	4.60562E−06
Epstein-Barr virus infection	10/85	231/9040	5.70522E−05
Herpes simplex virus 1 infection	14/85	459/9040	8.53326E−05
NOD-like receptor signaling pathway	9/85	216/9040	0.000183236
Human papillomavirus infection	10/85	362/9040	0.002015837
Toxoplasmosis	5/85	110/9040	0.003690371
p53 signaling pathway	4/85	72/9040	0.004608639
JAK-STAT signaling pathway	6/85	171/9040	0.005349247
Osteoclast differentiation	5/85	128/9040	0.006996706
Pyrimidine metabolism	3/85	56/9040	0.015545426
Necroptosis	5/85	176/9040	0.024823641
Mitophagy - animal	3/85	68/9040	0.025943081
RIG-I-like receptor signaling pathway	3/85	71/9040	0.029002581
Cytosolic DNA-sensing pathway	3/85	75/9040	0.0333668
Pancreatic cancer	3/85	76/9040	0.034508444
Platinum drug resistance	3/85	80/9040	0.039275685
Nucleotide metabolism	3/85	84/9040	0.044360598
PI3K-Akt signaling pathway	7/85	359/9040	0.051547108
Various types of N-glycan biosynthesis	2/85	40/9040	0.054134727
Ferroptosis	2/85	40/9040	0.054134727
Drug metabolism - other enzymes	3/85	92/9040	0.055461179
Small cell lung cancer	3/85	93/9040	0.056934027
Hematopoietic cell lineage	3/85	94/9040	0.05842545
Kaposi sarcoma-associated herpesvirus infection	5/85	224/9040	0.059482227
Transcriptional misregulation in cancer	5/85	224/9040	0.059482227
Viral carcinogenesis	5/85	229/9040	0.064160806
Hepatitis B	4/85	163/9040	0.067169568
Ras signaling pathway	5/85	235/9040	0.070043168
N-Glycan biosynthesis	2/85	50/9040	0.080104495
Hepatocellular carcinoma	4/85	174/9040	0.081046806
ABC transporters	2/85	52/9040	0.085687203
C-type lectin receptor signaling pathway	3/85	112/9040	0.088298834
Tuberculosis	4/85	180/9040	0.089173509
Sulfur metabolism	1/85	11/9040	0.098753363
Malaria	2/85	57/9040	0.100131191
Parkinson disease	5/85	264/9040	0.102516201
AMPK signaling pathway	3/85	127/9040	0.117164497

### Tanshinone IIA distribution in the brain

In order to obtain a comprehensive understanding of the distribution of Tanshinone IIA in the brain, we selected the sagittal plane of mouse brain tissue as an analytical sample and applied mass spectrometry imaging technology to spatially visualize the sliced samples, and we superimposed the optical images with the mass spectrometry imaging maps, accurate circling, and extraction of mass spectral data for each functional microtome (Figure 4F). According to the fragmentation pattern of Tanshinone IIA in negative ion mode, m/z 373.0733 was chosen as the observation ion, and we found that Tanshinone IIA was mainly distributed in the third ventricle (V3 third ventricle), the fourth ventricle (V4 fourth ventricle), the superior cerebellar peduncles (decussation of superior cerebellar peduncles), etc. Based on the results, it is further proved that Tanshinone IIA is able to cross the blood-brain barrier. This is the first time that the distribution of Tanshinone IIA in the brain has been examined at the spatial metabolome level. The study of the accurate distribution of Tanshinone IIA in the brain area will help in the precise dosing during the treatment process and is important for the study of the core target of Tanshinone IIA in neuroinflammation.

### Tanshinone IIA attenuates neuroinflammation independent of the UDP-GlcNAc pathway

The various endogenous metabolites are intricately linked between the different microregions of the brain and together regulate complex activities in the brain through metabolic pathways and neural networks. Tanshinone IIA treatment significantly altered the purine metabolic profile in the neuroinflammation model (Figure S4A). Specifically, Tanshinone IIA treatment decreased the levels of guanosine monophosphate and adenosine diphosphate (ADP), while increasing adenosine monophosphate (AMP), adenosine, and adenine. Levels of the downstream metabolites hypoxanthine and xanthines were also significantly reduced.

Given that targeting glycan metabolic pathways is a promising therapeutic strategy,^38^ we investigated the effect of Tanshinone IIA on the hexosamine biosynthesis pathway (HBP). The HBP synthesizes UDP-GlcNAc,^52^ the essential substrate for protein glycosylation. We found that Tanshinone IIA treatment significantly changed cellular UDP-GlcNAc levels (Figure S4B), initially suggesting it might counter neuroinflammation by inhibiting glycosylation. To test this hypothesis, we supplemented primary microglia with exogenous UDP-GlcNAc. However, this intervention failed to reverse the anti-inflammatory activity of Tanshinone IIA (Figure S4C), indicating that the anti-inflammatory mechanism of Tanshinone IIA operates independently of UDP-GlcNAc regulation.

### Analysis of the glycoproteome after Tanshinone IIA treatment

**Figure 5:**
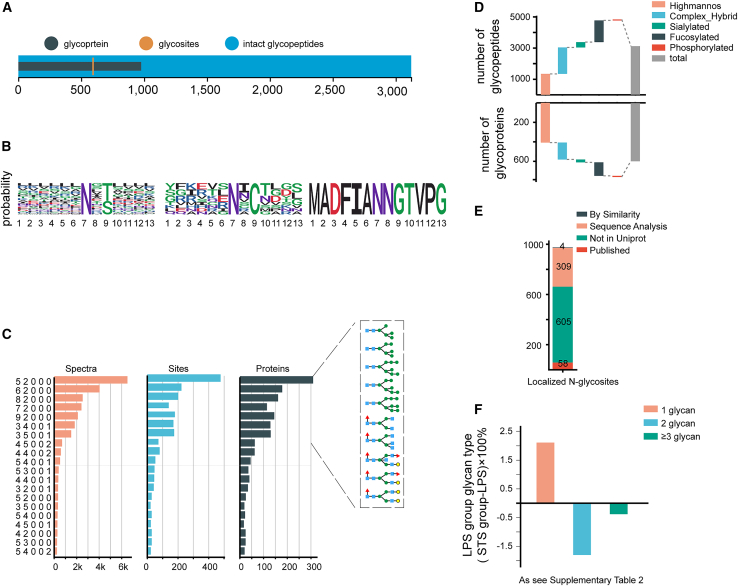
Tanshinone IIA modulates protein glycosylation levels (A) Enriched 599 glycoproteins; 976 glycosylation sites; and 3,122 intact glycopeptides. (B) Sequence motif of N-glycosite visualized by WebLogo. (C) Top 20 glycans, hexose (Hex, H), hexosamine (HexNAc, N), fucose (Fuc, F), acetylneuraminic acid (NeuAc, A), and glycolylneuraminic acid (NeuGc, G). (D) Different glycoforms correspond to the numbers of glycopeptides and glycoproteins. (E) Glycosites recorded in the Uniprot database. (F) Percentage of glycan content attached to glycosylation sites, vertical coordinate is (STS group - LPS group) × 100%. The glycan symbols are as follows: green circle for Hex, yellow circles for pentoses, blue square for HexNAc, purple diamond for sialic acids, and red triangle for fucose.

Following Tanshinone IIA treatment in the murine neuroinflammatory model, we performed a further glycoproteomic analysis. This analysis identified 588 glycoproteins; 976 glycosylation sites; 3,122 intact glycopeptides, and 264 glycans (Figure 5A). The N-glycoprotein sequence motifs were visualized using WebLogo (Figure 5B), and included NXS/T-type, NXC-type, and NXG-type. Additionally, the 20 most abundant glycan structures, all of which are oligosaccharides or complex N-glycans, are presented in Figure 5C. High-mannose and complex/hybrid types comprised the majority of glycopeptides and glycoproteins (Figure 5D), consistent with previous findings by Fang et al.^53^ Comparison of our N-glycoproteome data with the UniProt database revealed that 58 identified N-glycosylation sites (58/976) were previously annotated (Figure 5E). These findings significantly expand the known glycoproteome and provide a wealth of new targets for future disease studies. The corresponding glycoproteins were subjected to GO enrichment, where biological process (BP) includes amyloid precursor protein metabolic process and molecular function (MF) includes amyloid-beta binding (Figure S5A). The results further suggest that glycosylation modifications of proteins have research potential in neuroinflammation-related diseases.

Tanshinone IIA treatment significantly altered the glycosylation landscape in the neuroinflammatory model, reducing the number of ≥2 glycans compared to the model group (Figure 5F; Table S2), that is, the number of glycans present at the same glycosylation site was reduced. This suggests that Tanshinone IIA can exert a glycosylation change effect.

Multivariate statistical analysis of significantly altered glycopeptides (Figure S5B) revealed distinct separation among the three groups (control, model, and Tanshinone IIA-treated), which was further supported by heatmap analysis demonstrating distinct clustering (Figure S5C). These results confirm significant alterations in glycopeptide levels between the groups, with good reproducibility within each group.

We constructed protein-protein interaction networks using STRING and visualized the biological processes involving glycoproteins using Cytoscape, revealing significant involvement of altered glycoproteins in neuroinflammation and affecting the neuroinflammatory process (Figures S6A and S6B). We observed that LPS-induced neuroinflammation led to significant alterations in both N- and O-linked glycosylation, which were largely reversed by Tanshinone IIA treatment (Figures S6C and S6D). Functional enrichment analysis revealed that these dysregulated glycoproteins are predominantly involved in pathways critical for neuronal development and function, such as the glutamatergic synapse pathway (Figures S6E–S6G; Table S3).

Our findings suggest that Tanshinone IIA attenuates protein glycosylation by changing glycan occupancy on proteins, that is, the number of glycans present at the same glycosylation site is reduced. Our research findings indicate that Tanshinone IIA attenuates protein glycosylation by reducing glycosylation occupancy on proteins. However, the specific core glycosylated proteins responsible for its anti-inflammatory effects require further investigation.

### Tanshinone IIA exerts its anti-inflammatory effects by modulating the glycosylation of Hsp90b1

**Figure 6:**
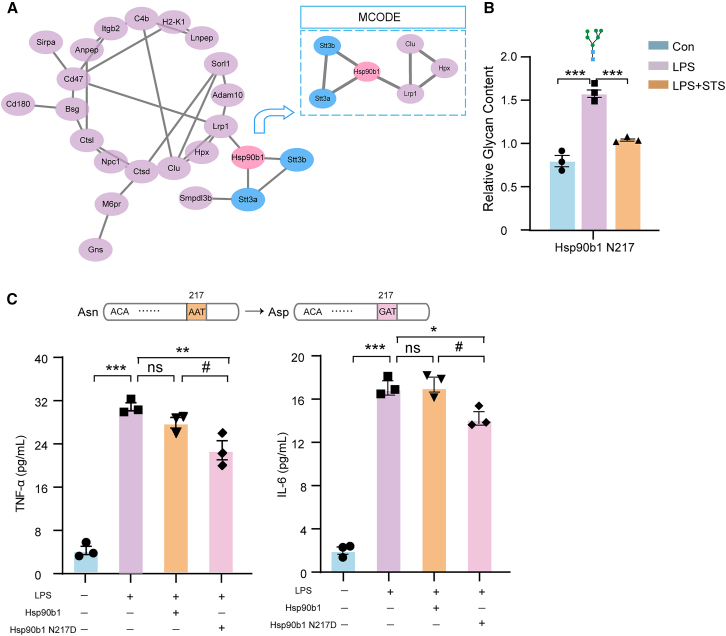
The Hsp90b1 mutation at position 217 suppresses neuroinflammation (A) The network analysis integrates differentially glycosylated proteins regulated by Tanshinone IIA with those modulated by the glycosylation inhibitor NGI-1, thereby identifying Hsp90b1 as a key protein commonly targeted by both NGI-1 and Tanshinone IIA. (B) Changes in the glycosylation level at position 217 of the Hsp90b1 protein in a neuroinflammatory model, with or without Tanshinone IIA treatment. Each group contained 3 samples. Error bars represent mean ± standard error (SE). Statistical analysis was performed by one-way ANOVA. *p* values indicating significance are depicted as follows: ∗*p* < 0.05, ∗∗*p* < 0.01, and ∗∗∗*p* < 0.001 vs*.* LPS group. (C) ELISA results showed the levels of TNF-α and IL-6 in the supernatant of primary microglia following the mutation of asparagine (Asn, N) at position 217 to aspartic acid (Asp, D) in the Hsp90b1 protein (i.e., Hsp90b1 N217D mutation). The glycan symbols are as follows: green circle for Hex and blue square for HexNAc. Each group contained 3 samples. Error bars represent mean ± standard error (SE). Statistical analysis was performed by two-way ANOVA. *p* values indicating significance are depicted as follows: ∗*p* < 0.05, ∗∗*p* < 0.01, and ∗∗∗*p* < 0.001, ns is non-significant vs*.* LPS group. ^#^*p* < 0.05, ^##^*p* < 0.01, and ^###^*p* < 0.001 vs*.* Hsp90b1 group.

To elucidate the key glycosylated proteins through which Tanshinone IIA exerts its anti-inflammatory effects, we constructed a protein-protein interaction network using the STRING database. This analysis integrated the differentially glycosylated proteins regulated by Tanshinone IIA with those affected by the N-linked glycosylation inhibitor, NGI-1. Our analysis identified Hsp90b1 as a key protein commonly targeted by both NGI-1 and Tanshinone IIA (Figure 6A). This finding is particularly significant given that Hsp90b1 is a known regulator of the pro-inflammatory NF-κB pathway.^54^ Specifically, we discovered that Tanshinone IIA modulates glycosylation at position 217 of Hsp90b1, thereby significantly attenuating the aberrant glycosylation induced by LPS (Figure 6B). These results strongly suggest that glycosylation at this specific site is a critical regulatory point in the inflammatory response.

To directly test this hypothesis, we proceeded to investigate the impact of site-specific mutations in Hsp90b1 on its ability to mediate inflammation. We introduced a site-directed mutation, specifically N217D, into Hsp90b1. Subsequently, the protein was expressed in cells, and inflammatory cytokine levels were measured. The results indicate that Hsp90b1 with the N217 mutation exhibits a significantly reduced ability to promote inflammation (Figure 6C).

Collectively, Tanshinone IIA exerts its anti-inflammatory effects by modulating the glycosylation of Hsp90b1 at position 217.

## Discussion

This study employed a quantitative proteomics approach to investigate the mechanisms of LPS-induced neuroinflammation in mice. While corroborating previous findings, we discovered significant regulation of proteins involved in neuroinflammation. Using the ZIC-HILIC-HCD-Orbitrap workflow, we performed unbiased, large-scale glycoproteomic profiling of brain tissue from neuroinflammatory mice, revealing the role of protein glycosylation in this process. Importantly, our proteomic data suggested that Tanshinone IIA, a potential therapeutic agent for neuroinflammatory diseases, exerts its effects by modulating glycosylation pathways.

LPS successfully induced neuroinflammation in both a mouse model and microglial cells, evidenced by increased mRNA and protein expression of pro-inflammatory cytokines, including TNF-α, IL-6, and IL-1β (Figures 1A and 1B; Figure 3E; Table 2). Proteomic analysis of the mouse model identified a set of dysregulated proteins involved in inflammation (Figures 1C–1F), which, intriguingly, were characterized as glycoproteins. Nxn, a negative regulator of the Wnt signaling pathway involved in inflammation, and Samhd1, an inhibitor of TAK1 and TRAF6 signaling,^30^ were also affected. Upregulations of IL1R1, the receptor for IL-1β, and Lpcat2, which is involved in PAF release,^32^ as well as Nfkb2, a key component of the non-canonical NF-κB pathway,^43^ were also observed. Consistent with previous reports,^44^^,^^45^^,^^46^ we found increased expression of Fyb1, Pik3ap1, Tnip1, CD14, Slpi, and Tlr2 (Figure S1E). We found that these dysregulated proteins associated with neuroinflammation are essentially glycoproteins. This finding provides further evidence that glycoproteins play a role in neuroinflammation.

**Table 2:** Summary of the RT-qPCR primers sequences

Gene	Primer	Sequence (5′ to 3′)
Mouse GAPDH	forward	TGACCTCAACTACATGGTCTACA
Mouse GAPDH	reverse	CTTCCCATTCTCGGCCTTG
Mouse TNF-α	forward	CAGGCGGTGCCTATGTCTC
Mouse TNF-α	reverse	CGATCACCCCGAAGTTCAGTAG
Mouse IL-6	forward	CTGCAAGAGACTTCCATCCAG
Mouse IL-6	reverse	AGTGGTATAGACAGGTCTGTTGG
Mouse IL-1β	forward	AGCTGGAGAGTGTGGATCCC
Mouse IL-1β	reverse	CCTGTCTTGGCCGAGGACTA

Our glycoproteomic analysis provided experimental evidence for over 300 predicted N-glycosylation sites in UniProt, confirmed nearly 60 known sites, and identified over 600 novel sites (Figure 5E). While the expression of inflammation-related proteins remained unchanged between control and neuroinflammatory groups, their glycosylation levels were significantly altered (Figure 2E). Specifically, N-glycosylation of Sort1, Slc1a2, Atp1b1, Nrcam, and Nptn was increased, while that of Lrp1, Thy1, Hyou1, L1cam, and Mag was decreased in the LPS group (Figure 2F). Similar trends were observed for O-glycosylation (Figure 2G). These glycoproteins play crucial roles in immunity and inflammation.^35^^,^^36^^,^^37^ LRP1 plays a crucial role in AD by regulating synaptic plasticity, insulin signaling, and lipid metabolism. Its absence induces chronic neuroinflammation and glucose metabolism disorders, accelerating cognitive decline. Additionally, reduced LRP1 expression impairs Aβ clearance, leading to increased intracerebral deposition and exacerbating neuronal damage.^55^

Furthermore, the proportion of proteins with ≥2 glycans attached to glycosylation sites was elevated in the neuroinflammation group (Figure 2H), indicating variations in protein glycosylation levels. Treatment of BV2 cells with NGI-1, an inhibitor of OST, attenuated LPS-induced increases in TNF-α, IL-6, and IL-1β (Figures 3E and 3F), supporting the hypothesis that modulating protein glycosylation can ameliorate neuroinflammation.^13^^,^^18^^,^^56^

Combining our proteomic and glycoproteomic analyses (Figure 4C), we observed that the most significantly altered proteins or glycosylated proteins are closely linked to the central neurodegenerative AD. These findings suggest that, firstly, inflammation plays a crucial role in the pathogenesis of degenerative diseases and warrants further investigation and secondly, modulating glycosylation modifications to inhibit inflammation may potentially slow the progression of AD. This hypothesis is strongly supported by existing literature. Previous studies have already documented global alterations in brain tissue glycan profiles and specific changes at glycosylation sites in AD patients.^14^ Notably, N-glycosylated tau, a hallmark protein of the disease, has been identified specifically in the brains of AD patients but not in healthy controls, directly linking aberrant glycosylation to AD-specific pathology.^15^ Thus, our findings not only align with these previous observations but also provide a specific pharmacological avenue—targeting glycosylation—to potentially intervene in the inflammatory cascade central to AD.

Connectivity Map analysis of our proteomic data identified Tanshinone IIA as a promising candidate for treating neuroinflammation (Figure 4A). Mass spectrometry imaging confirmed its ability to cross the blood-brain barrier, accumulating primarily in the third and fourth ventricles and the superior cerebellar peduncle (Figure 4F). We also verified that Tanshinone IIA reduced the expression of TNF-α, IL-6, and IL-1β *in vitro* and *in vivo* (Figures S2D and S2E).

This study provides the first evidence that Tanshinone IIA’s anti-neuroinflammatory effects are mediated by modulation of protein glycosylation. Tanshinone IIA treatment altered LPS-induced changes in N-glycan biosynthesis pathways (Table 1) and changed glycan levels (Figure 5H). Through a comparative proteomic analysis integrated with a protein-protein interaction network, we identified Hsp90b1 (a crucial regulator of the pro-inflammatory NF-κB signaling pathway) as a key common target for Tanshinone IIA (Figure 6A). Moving beyond this initial identification, we demonstrated that Tanshinone IIA specifically modulates the glycosylation of Hsp90b1 at asparagine 217 (N217), effectively reversing the aberrant glycosylation induced by LPS (Figure 6B). Crucially, the functional significance of this site-specific modification was confirmed through site-directed mutagenesis. The N217D mutant of Hsp90b1 exhibited a markedly diminished capacity to promote inflammation, providing direct evidence that glycosylation at this precise locus is essential for its pro-inflammatory activity (Figure 6C). Together, these findings delineate a specific mechanism: Tanshinone IIA alleviates neuroinflammation by selectively targeting and inhibiting the glycosylation of Hsp90b1 at the N217 site, thereby disrupting a key regulatory node in the inflammatory cascade. Furthermore, we posit that the interplay among Tanshinone IIA, glycosylation modification, and neuroinflammation constitutes a sophisticated regulatory response orchestrated by multi-layered molecular interactions. Given the extensive glycoproteomic alterations observed, the specific Hsp90b1 locus represents merely one pivotal node within this intricate regulatory network, and the underlying mechanisms remain to be elucidated through in-depth investigation.

Our study demonstrates that Tanshinone IIA inhibits neuroinflammation by suppressing protein glycosylation, a mechanism shared with the known inhibitor NGI-1. However, their modes of action diverge significantly. NGI-1 is a global glycosylation inhibitor that directly targets the OST enzymatic complex.^57^^,^^58^ In contrast, Tanshinone IIA achieves its anti-inflammatory effect through a more targeted mechanism: it selectively inhibits the glycosylation of the NF-κB-interacting protein Hsp90b1 at a specific site.

Tanshinone IIA effectively attenuates neuroinflammation while simultaneously reducing cerebral UDP-GlcNAc levels and global protein glycosylation, characterized by decreased glycan occupancy and altered glycoproteomic landscapes (Figure 5; Figures S2 and S4). Given that UDP-GlcNAc is the indispensable substrate for N-linked glycosylation,^59^ we initially hypothesized that Tanshinone IIA exerts its anti-inflammatory effects via substrate-level depletion. However, our observation that exogenous UDP-GlcNAc supplementation failed to reverse the anti-inflammatory activity in primary microglia (Figure S4C) suggests a more complex regulatory mechanism beyond simple substrate availability. Our findings pinpoint the glycosylation of Hsp90b1 at the N217 site as a critical node in Tanshinone IIA’s mechanism (Figure 6). Hsp90b1 (GRP94), an ER-resident member of the HSP90 family, functions as a specialized chaperone essential for the folding, glycosylation, and quality control of nascent glycoproteins.^60^^,^^61^ We propose that the Tanshinone IIA-induced reduction in N217 glycosylation on Hsp90b1 impairs its chaperone capacity. This dysfunction, in turn, leads to the widespread alterations observed in the glycoproteomic landscape, as Hsp90b1 is required to provide the structural scaffold for the correct processing of inflammatory client proteins. While the exact cause of the parallel reduction in UDP-GlcNAc remains to be fully elucidated, our data suggest that the therapeutic efficacy of Tanshinone IIA is primarily driven by the targeted disruption of Hsp90b1-mediated protein maturation rather than global substrate exhaustion. Unlike global glycosylation inhibitors, which induce massive ER stress by broad substrate deprivation, Tanshinone IIA selectively targets the glycosylation status of Hsp90b1, thereby fine-tuning the maturation of specific pro-inflammatory clients. This chaperone-centric mechanism provides a more targeted therapeutic window for neuroinflammation.

In summary, this study pioneers the investigation of the role of protein glycosylation in neuroinflammation through a quantitative proteomics and glycoproteomic approach. In this study, we identified novel glycosylation sites and characterized their alterations during LPS-induced neuroinflammation. We further demonstrated that Tanshinone IIA exerts its anti-neuroinflammatory effects by targeting these aberrant glycosylation events. Mechanistically, we revealed that the inhibition of Hsp90b1 glycosylation at the N217 residue is a critical event mediating its therapeutic efficacy. Meanwhile, integration analysis of proteome and glycoproteome suggests that neuroinflammation plays a crucial role in the pathogenesis of degenerative diseases and warrants further investigation. Our study provides a new therapeutic avenue for neuroinflammation by targeting glycosylation modification, and it provides a potential strategy for treating neurological diseases arising either due to congenital disorders of glycosylation or other neurological disorders.

### Limitations of the study

This study has several limitations. First, our investigation was confined to the mechanisms of neuroinflammation and did not extend to the specific neurodegenerative diseases it precipitates. Future research is required to translate these findings into precision therapies for distinct pathological conditions. Furthermore, while inhibiting protein glycosylation effectively alleviated inflammation, this global suppression carries the risk of significant off-target effects. A more refined and safer anti-inflammatory strategy would involve selectively targeting the glycosylation of key proteins within the inflammatory cascade. This reciprocal regulation between glycation and neuroinflammation complicates efforts to disentangle their causal dependencies, necessitating further research to determine the specific causal mechanisms. Finally, it is crucial to acknowledge that the acute LPS-induced neuroinflammation model used herein may not fully recapitulate the chronic, low-grade inflammatory processes characteristic of neurodegenerative diseases.

## Resource availability

### Lead contact

Requests for further information and resources should be directed to and will be fulfilled by the lead contact, Dezhi Kong (kongdezhi@hebmu.edu.cn).

### Materials availability

This study does not generate new, unique reagents.

### Data and code availability

• All data reported in this article are available from the lead contact upon request. The mass spectrometry proteomics data have been deposited to the ProteomeXchange Consortium (https://proteomecentral.proteomexchange.org) via the iProX partner repository^62^^,^^63^ with the dataset identifier PXD065987 and PXD066194.
• No original code is reported in this work.
• Any additional information regarding the sources or analyses of the data reported here may be obtained from the lead contact upon request.

## Acknowledgments

We gratefully acknowledge the financial support provided by Qiuhong Guo for this research.

This study received partial financial support in the form of the 10.13039/501100001809National Natural Science Foundation of China (grant nos. 82474103, 82174004, and 82471479), the Hengrui Hebei Innovation and Development Medical Cooperation Programme Project (grant no. HR202502096), the National Science Foundation of Hebei Province (grant no. H2023423003), and the 10.13039/501100012166National Key Research and Development Program of China (grant no. 2022YFC3500501).

## Author contributions

Y.L., original draft, investigation, and data curation; K.D., conceptualization and resources; G.L., validation and visualization; J.Y., data curation and validation; Q.W., validation and visualization; Y.Z., methodology; Y.W., validation; B.G., data curation; P.Z., data curation; W.Z., conceptualization, project administration, and review; and D.K., conceptualization, supervision, funding acquisition, and review.

## Declaration of interests

The authors declare no competing interests.

## STAR★Methods

### Key resources table

**Table:** 

REAGENT or RESOURCE	SOURCE	IDENTIFIER
**Bacterial and virus strains**		

DH5α	Transgen Biotech	Cat#:CD201

**Biological samples**		

Mouse cortex	This paper	N/A

**Chemicals, peptides, and recombinant proteins**		

DMEM	Biological Industries	Cat#: C3113
FBS	Thermo Fisher Scientific	Cat#: A5256701
Lipomaster3000	vazyme	Cat#: TL301
Triethylammonium bicarbonate	Sigma-Aldrich	Cat#: T7408
Iodoacetamide	Sigma-Aldrich	Cat#: V900335
Trifluoroacetic acid	Sigma-Aldrich	Cat#: 302031
TRI RNA lysis solution	ZHONGSHI TONTRO	Cat#: ZS-M11008
Methanol (MeOH)	Thermo Fisher Scientific	Cat#: 022909-K7
Sulfotanshinone Sodium Injection	Shanghai First Biochemical Pharmaceutical Co., Ltd.	N/A
Acetonitrile (ACN)	Thermo Fisher Scientific	Cat#: 047138-M1
TPCK-Trypsin	Thermo Fisher Scientific	Cat#: 20233
Formic acid	Thermo Fisher Scientific	Cat#: 28905
Dithiothreitol	Sangon Biotech	Cat#: A620058
Grade trypsin	Promega	Cat#: V5111
NGI-1	AbMole	Cat#: M9829

**Critical commercial assays**		

Cell Counting Kit-8	Reprot	Cat#: RK1028
PrimeScript™ RT Reagent Kit with gDNA Eraser	Takara	Cat#: RR047A
MonAmp™ ChemoHS qPCR Mix	Monad	Cat#: MQ00401
Mouse Tumor Necrosis Factor Alpha (TNF-a) ELISA Kit	JONLNBIO	Cat#: JL10484
Mouse Interleukin 6 (IL-6) ELISA Kit	JONLNBIO	Cat#: JL20268
Mouse Interleukin 1 Beta (IL-1β) ELISA Kit	JONLNBIO	Cat#: JL18442

**Deposited data**		

Raw data from mass spectrometry proteomics	iProX	PXD065987
Raw data from mass spectrometry glycoproteomics	iProX	PXD066194

**Experimental models: Cell lines**		

BV2 cells	Shanghai Institutes for Biological Sciences	Cat#: GNM45
Primary microglia cells	SSRCC	Cat#: JY-J659
HEK293T cells	Shanghai Institutes for Biological Sciences	Cat#: SCSP-5209

**Experimental models: Organisms/strains**		

Mouse model of neuroinflammation	This paper	N/A

**Oligonucleotides**		

Primers for protein expression mutations	This paper	Table S4
Primers for protein expression qPCR	This paper	Table 2

**Recombinant DNA**		

pcDNA3.1-T2A-EGFP	MiaoLing Plasmid	N/A

**Software and algorithms**		

DIA-NN	Github	https://github.com/vdemichev/DiaNN
pGlyco 3.0	Github	https://github.com/pFindStudio/pGlyco3/releases
Xcalibur 3.0	Thermo Fisher Scientific	N/A
GraphPad Prism 8.0.2	GraphPad	https://www.graphpad.com/resources
GSEA_4.3.2	GSEA	https://www.gsea-msigdb.org/gsea/index.jsp
ImageJ 1.54	ImageJ	https://imagej.nih.gov/ij/

**Other**		

Nano-scale ultra-high-performance liquid chromatograph	Thermo Fisher Scientific	Easy-nLC 1000
Orbitrap mass spectrometer	Thermo Fisher Scientific	Orbitrap Fusion
NanoDrop	Thermo Fisher Scientific	NanoDrop 2000
Multifunctional Microplate Reader	BMG Labtech	Infinite 200 Pro Multifunctional Microplate Reader
Refrigerated High-Speed Centrifuge	Thermo Fisher Scientific	Cat#: 75002447
ZIC-HILIC	Merck	20 × 2.1 mm, 5 μm

### Experimental model and study participant details

#### Animals

Wild-type (WT) male C57BL/6J mice aged 8 weeks (weight, ∼20 g) were purchased from Hebei *Invivo* Biotechnology Co., Ltd (Shijiazhuang, China) and housed at the Laboratory Animal Resource Center of Hebei Medical University. They were bred and maintained under pathogen-free conditions with free access to food and water. All animal experiments complied with ARRIVE guidelines. All procedures were in compliance with ethical regulations and approved by the ethics committee of Hebei medical university (ethical approval number: IACUCC-Hebmu-2023026). Only male mice were used in this study to minimize variability related to sex differences. No sex-specific effects were evaluated in this study.

The experiment is conducted in batches, including model establishment and drug treatment. The mice were randomly divided into three groups: vehicle group (0.9% NaCl, i.p.), the neuroinflammation model group (LPS, 2 mg/kg, i.p.) and Tanshinone IIA group (2 mg/kg LPS +10 mg/mL Tanshinone IIA). In this case, the Tanshinone IIA group received an intraperitoneal injection of Tanshinone IIA half an hour after LPS administration.

Mice were euthanized by cervical dislocation. The brain tissues were quickly removed after the perfusion with ice-cold 0.9% NaCl. Then, the tissues were snap frozen in liquid nitrogen and stored at −80°C until use. All procedures were in compliance with ethical regulations and approved by the ethics committee of Hebei medical university.

The cerebral cortex was divided into 3 sections for proteomic experiments, RT-qPCR, and ELISA assays, respectively.

#### Cell culture and harvest

BV2 cells (murine microglia, SCSP-5208) and HEK293T cell (SCSP-502) were purchased from National Collection of Authenticated Cell Cultures (Shanghai, China). These cells were cultured at 37°C with 5% CO_2_ in DMEM containing 10% fetal bovine serum, 100 μg/mL streptomycin, and 100 U/mL penicillin.

Primary microglia cells culture was performed as previously described.^64^ Briefly, cortexes were removed aseptically from the skulls and meninges were excised carefully under a dissecting microscope. Primary glial cells were obtained from the cerebral cortices, which were earlier digested by 0.25% Trypsin/EDTA at 37°C for 20 min and seeded into 0.01% poly-lysine-coated (PLL) 75 cm^2^ culture flasks. The cultures were maintained for 2 weeks in complete DMEM. Media was replaced one day after preparation and subsequently every 2–3 days. After mixed glial cultures were completely confluent, microglia cells were separated by shaking at 180 rpm on a gyratory shaker for 30 min at 37 °C and then reseeded in culture dishes for subsequent research. The percentage of the primary microglia cells was confirmed by CD68 staining with over 97% purity.

Authentication of all cell lines was performed. BV2 cell and HEK293T cell lines were used for short tandem repeat (STR) profiling. The percentage of the primary microglia cells was confirmed by CD68 staining with over 97% purity. The results of mycoplasma testing for BV2 cells and HEK293T cells were negative. Primary microglia were not tested for mycoplasma.

### Method details

#### Materials and reagents

LC/MS-grade methanol (MeOH), acetonitrile (ACN), TPCK-Trypsin and formic acid were purchased from Thermo Fisher Scientific (Waltham, MA, USA). Deionized water was prepared by a Thermo Nanopure water purification system (Waltham, MA, USA). Triethylammonium bicarbonate (TEAB), iodoacetamide (IAM), trifluoroacetic acid (TFA) and ammonia were purchased from Sigma-Aldrich (Shanghai, China). Dithiothreitol (DTT) was purchased from Sangon Biotech Co. (Shanghai, China). Ammonium formate was obtained from Tianjin Yongda Chemical Reagent Co. (Tianjin, China). Grade trypsin was purchased from Promega (Madison, Wisconsin, USA). The FBS was purchased from Thermo Fisher Scientific Australia Pty. Ltd. (Thornton, Australia). The high glucose DMEM media, phosphate buffered saline, and 0.25% trypsin EDTA were obtained from Biological Industries (Shanghai, China). The cell counting Kit-8 (CCK-8) was obtained from Report Bio & Technology Co. Ltd. (Shijiazhuang, China). TRI RNA lysis solution was purchased from Zhongshi Gene Technology Co., Ltd (Tianjing, China). The first strand cDNA synthesis kit was brought from Takara Bio Inc. (Beijing, China). MonAmpTm ChemoHS qPCR Mix were purchased from Monad Biotechnology Co., Ltd. (Suzhou, China). The Sulfotanshinone Sodium Injection were purchased from SHP NO.1 Biochemical and Pharmaceutical Co., Ltd (Shanghai, China). The ELISA kits were purchased from Shanghai future industrial Limited by Share Ltd (Shanghai, China). ZIC-HILIC (20 × 2.1 mm, 5 μm) was purchased from Merck KGaA (Darmstadt, Germany). The pcDNA3.1-Hsp90b1-T2A-EGFP was purchased from MiaoLing Plasmid (Changsha, China). CD68 was purchased fromBioss Antibodies (Woburn, MA, USA). All other reagents were of analytical purity.

#### Cell viability assay

Cells were seeded in 96-well plates (5×10^3^ cells/mL), cultured in medium for 24 h, treated with different concentrations of LPS solution (0.1, 0.5, 1.0, 1.5, 2.0, and 3.0 μg/mL), NGI-1 solution (2.5, 5, 10, 25 and 50 μM) and Tanshinone IIA solution (0, 200, 400, 600, 800, 1000, and 2000 μM), and incubated for 24 h. Then, CCK-8 was added to each well. The optical density of each well at 450 nm was measured on a microplate reader (Thermo Fisher Scientific, Waltham, USA), and cell viability was calculated. Data were graphically displayed using GraphPad Prism (Version 8.0). In the end, 1.0 μg/mL LPS, 200 μM and 300 μM Tanshinone IIA were chosen as the delivery concentrations.

#### RT-qPCR

RT-qPCR was used to determine the mRNA level of IL-6 and TNF-α in the cerebral cortex and cells. Total RNA was purified using the Trizol. The cDNA was synthesized using a PrimeScript RT Reagent Kit with gDNA Eraser. RT-qPCR was performed using MonAmp ChemoHS qPCR Mix according to the manufacturer’s instructions. The primer sequences are listed in Table 2. The relative expression of TNF-α, IL-6 and IL-1β mRNA to GAPDH mRNA was determined using the ^ΔΔ^Ct method.

#### Determination of TNF-α, IL-1, and IL-6 in cerebral cortex

Inflammatory cytokines levels, including TNF-α, IL-1β, and IL-6 levels in the cerebral cortex, were determined using commercial ELISA kits. All commercial kits were used according to the manufacturer’s instructions.

#### Preparation of mouse tissue samples

50 mg pieces of frozen tissues were homogenized in 0.4 mL of 8 M urea buffer (8 M UA in 50 mM Tris/HCl, pH 8.0) by a high-throughput tissue grinding machine (ONEBIO, China) at 60 Hz for 60 s. After centrifugation at 10,000 × *g* at 4°C for 10 min, the supernatants were sonicated with the aid of Cell Sonicator (Ningbo, China) in an ice bath for 60 s. Protein concentrations for each sample were determined by BCA method after centrifugation at 12,000 ×*g* at 4°C for 10 min.

#### Protein digestion

The protein samples were reduced by dithiothreitol (20 mM) for 1 h at 37°C, followed by alkylation with iodoacetamide (40 mM) at 25°C for 45 min in the dark. Six volumes of methanol were added to precipitate the proteins at −40°C for at least 4 h. The precipitates were dissolved in a digestion buffer (50 mM NH_4_HCO_3_). The protein samples were digested by adding trypsin at an enzyme/substrate ratio of 1:50 (*w/w*) for 12 h at 37°C. The reaction was terminated by the addition of 2% trifluoroacetic acid in 8% ACN. The peptides were desalted with C18 solid-phase extraction (SPE) cartridges. Eluted peptides were dried under vacuum in a lyophilizer and stored at −40°C for use.

#### Glycopeptides enrichment

The protein samples were digested by adding trypsin at an enzyme/substrate ratio of 1:20 (*w/w*) for 12 h at 37°C. The reaction was terminated by the addition of 2% trifluoroacetic acid. The peptides were desalted with C18 solid-phase extraction (SPE) cartridges. Samples were dissolved in 500 μL of equilibrium solution (0.1% TFA in 80% ACN). The ZIC-HILIC enrichment column was connected to an Agilent Technologies 1260 and activated for 5 min using 0.1% TFA in 2% ACN buffer solution at a flow rate of 1 mL/min, after which it was fully activated for 30 min using a flow rate of 0.1 mL/min, the enrichment column was equilibrated with 5 mL (at a flow rate of 1 mL/min) of an equilibration solution of 0.1% TFA in 80% ACN, after which the column was fully equilibrated with 0.1% TFA in 80% ACN for 30 min (flow rate 0.1 mL/min). After that, the sample dissolved in the equilibrium solution was loaded onto the enrichment column that had been fully activated and equilibrated, and the sample loading was repeated 3 times, and finally, the enrichment column was fully eluted using 0.1% TFA in 2% ACN at a flow rate of 0.2 mL/min for 5 min. The elution solution was collected, dried by centrifugation, and stored at −80°C.

#### Preparation and processing of brain tissue section

Mice were executed by decapitation, and the intact brain tissues were isolated and rinsed with ice-cold saline to eliminate residual surface blood. After draining excess liquid with filter paper, the brain tissues were placed in light-proof aluminum foil boxes and immediately snap-frozen in liquid nitrogen. All tissue samples were stored at −80°C prior to sectioning.

Brain tissues were sectioned into 10 μm serial frozen sections at −20°C using a cryostat microtome. Two sets of tissue sections were mounted onto SUPERFROST PLUS slides (Thermo Fisher Scientific, Bremen, Germany) for AFADESI-MSI. Prior to AFADESI-MSI analysis, the tissue sections were dried under vacuum for approximately 15 min. Imaging of tissue metabolites was performed using a custom-built AFADESI-MSI platform equipped with an Orbitrap mass spectrometer (Thermo Fisher Scientific, Bremen, Germany) and an AFADESI ion source. The experiments were conducted in both positive and negative ion modes across a mass-to-charge ratio (m/z) range of 100–1000. The spray solvent consisted of acetonitrile and water (80:20, *v/v*), with a flow rate set to 5.0 μL/min. The sprayer voltage was adjusted to −4500 V in negative ion mode, and the extraction gas flow for the AFADESI ion source was maintained at 12 L/min. The nebulizing gas (N_2_) flow rate was set to 0.6 MPa. Imaging analysis involved continuous scanning of the tissue section in the x-direction at a speed of 150 μm/s, with a vertical step size of 100 μm in the y-direction.

#### Nano-liquid chromatography-mass spectrometry for peptides analysis

Desalted peptides were reconstituted in 10 μL of 0.1% formic acid in water and analyzed using data-independent acquisition (DIA) on an Easy-nLC 1000 system coupled to an Orbitrap Fusion mass spectrometer. For peptide ionization, 1800 V was applied, and a 320°C capillary temperature was used. For MS detection, a precursor scan was performed in the Orbitrap by scanning from m/z 350–1250 with a resolution of 60000, a targeted automatic gain control (AGC) value of 4.0e^5^, and a maximum injection time of 50 ms. The ions selected under top-speed mode were isolated with quadrupole and they were fragmented by higher energy collision-induced dissociation (HCD) with a normalized collision energy of 35%, then detected in the Orbitrap. The typical MS/MS scan conditions were as follows: the Orbitrap resolution of 3000, a targeted AGC value of 1.0e^5^. A set of 38 windows of variable width was constructed with the mass range from 375 to 1015 m/z for Data-independent acquisition (DIA) study.

#### Nano-liquid chromatography-mass spectrometry for intact glycopeptides analysis

Data-dependent acquisition (DDA) was utilized on an Easy-nLC 1000 system coupled to the Orbitrap Fusion mass spectrometer. Samples were loaded onto a C18 column (20 cm × 75 μm i.d., 3 μm) and separated at a flow rate of 300 nL/min. The solvents used were 0.1% formic acid in water (solvent A) and acetonitrile containing 0.1% formic acid (solvent B). The gradient for glycoprotein mixtures was as follows: 2–4% solvent B for the first 5 min, 4–25% solvent B over 140 min, increasing to 35% solvent B in 20 min, followed by a ramp to 95% solvent B in 10 min, and finally holding at 100% solvent B for 5 min.

The MS parameters for glycopeptide analysis were set as follows: (1) Ion Source Type = NSI; Ion Source Tube Temp = 300°C; Positive Ion = 1800 V; Internal Cal Positive m/z = 445.12003; (2) MS: scan range (m/z) = 350–2000; RF lens (%) = 60; AGC target = 500,000; resolution = 120,000; included charge state is 2–6; maximum injection time is 50 ms; dynamic exclusion after n times, *n* = 1; exclusion duration (s) = 30; each selected precursor was subject to one HCD-MS/MS; (3) HCD-MS/MS: precursor selection range (m/z) = 700–2000; Isolation Mode = Quadrupole; isolation window = 4; Orbitrap resolution = 15,000; AGC target = 5.0 × 10^5^; maximum injection time = 250 ms; stepped collision mode on, collision energy = 30%, energy difference of ±10%; Date Type = Centroid.

#### Data analysis of peptides

DIA-NN, a specialized software tool for analyzing complex data from DIA mass spectrometry. It features automatic retention time alignment, which enhances data processing efficiency. Searches were conducted under the following parameters: (1) allowance for 2 missed cleavages; (2) incorporation of modifications including cysteine carbamidomethylation, N-terminal methionine excision, N-terminal acetylation, and oxidation; (3) a peptide length range set from 7 to 30 amino acids; (4) a precursor charge range of 1–4; (5) a precursor m/z range of 300–1800, with a false discovery rate (FDR) threshold of 1%.

#### Glycopeptide data analysis

For glycopeptide identification, the following parameters were applied: precursor mass tolerance set to 10 ppm and fragment ion tolerance to 20 ppm. A maximal of 2 missed cleavage was allowed. Fixed modifications included carbamidomethylation of all Cys residues (C: +57.022 Da). Besides, we used 3 variables modifications: oxidation (M: +15.995 Da), acetylation on the N-terminus (+42.0106 Da), and deamidation (N, Q: +0.984 Da). Raw data were analyzed using pGlyco 3.0, employing default parameters unless specified otherwise, with an FDR of 1% applied to glycopeptide-spectrum matches (GPSMs). The pGlyco 3.0 software filters out non-glycopeptide spectra by identifying glycopeptide-diagnostic ions and subsequently searches for glycan structures by indexing ions associated with glycans. Precursor ion tolerance was set to 10 ppm, while fragment ion tolerance was set to 20 ppm.^65^^,^^66^ Additionally, glycosylated proteome identification and analysis were conducted using GlycAP (https://project.omic solution.com/GlycAP/?QA).

#### AFADESI-MSI data analysis

The raw data file Raw format was converted to cdf format using Xcalibur 3.0 (Thermo Fisher Scientific, USA) to prepare the data files for mass spectral imaging. The converted data were then imported into the MassImager 1.0 workstation for data reading, ion image reconstruction, and background subtraction. Adjacent tissue sections within the region of interest were selected by overlaying optical images, followed by mass spectrometry imaging to obtain an average mass spectrum of the area. The resulting 2D matrix data were saved in.txt format and imported into MarkerView 1.2.1 (ABSCIEX, USA) for peak alignment and isotope ion removal, applying a mass tolerance of 5 ppm.

#### Glycosylation site mutation

The N-glycosylation site at position 217 was mutated from the asparagine amino acid sequence (AAT) to the aspartic acid sequence (GAT) (Table S4). This sequence was cloned into the pcDNA3.1-T2A-EGFP vector to form a recombinant plasmid for subsequent experiments.

#### Bioinformatics analysis and statistical analysis

Data visualization was performed using the online platform Microbiology Letter (http://www.bioinformatics.com.cn/). Proteomic data underwent statistical analysis via the Wukong: Quick View and Analyze Data cloud platform (https://www.omicsolution.org/wkomics/main/). The biological functions of proteins were explored using the CluGO plug-in within Cytoscape software, while protein-protein interactions were illustrated using STRING (https://cn.string-db.org/) in conjunction with Cytoscape.

For statistical analysis, Prism software ver. 8.0.2 (Graph-Pad, La Jolla, CA, USA) was used. To compare data between two independent groups, we used a *t* test. To compare data between three independent groups, we used a one-way ANOVA. two-way ANOVA was applied to compare data and evaluate the interaction between factors. The data are expressed as mean ± Standard error, and the differences were considered statistically significant at *p* < 0.05.
