# Supplementary material for: Glycosylation modulation as a therapeutic strategy for neuroinflammatory disorders: The potential of Tanshinone IIA
Source: iScience. 2026 May 20;29(6):116012. doi: 10.1016/j.isci.2026.116012 (PMC13214265; doi:10.1016/j.isci.2026.116012)
Supplement: Document S1. Figures S1–S6 and Tables S1–S4 [file mmc1.pdf]

iScience, Volume *29*

Supplemental information

Glycosylation modulation as a therapeutic strategy for neuroinflammatory disorders: The potential of Tanshinone IIA

Yahui Li, Kunfeng Duan, Guangyuan Liu, Jingwen Yan, Qian Wang, Yuyu Zhang, Yuran Wang, Bowen Guo, Panpan Zhang, Wei Zhang, and Dezhi Kong

Figure S1 Proteome-wide profiling of LPS-induced mouse brains and BV2 cells.

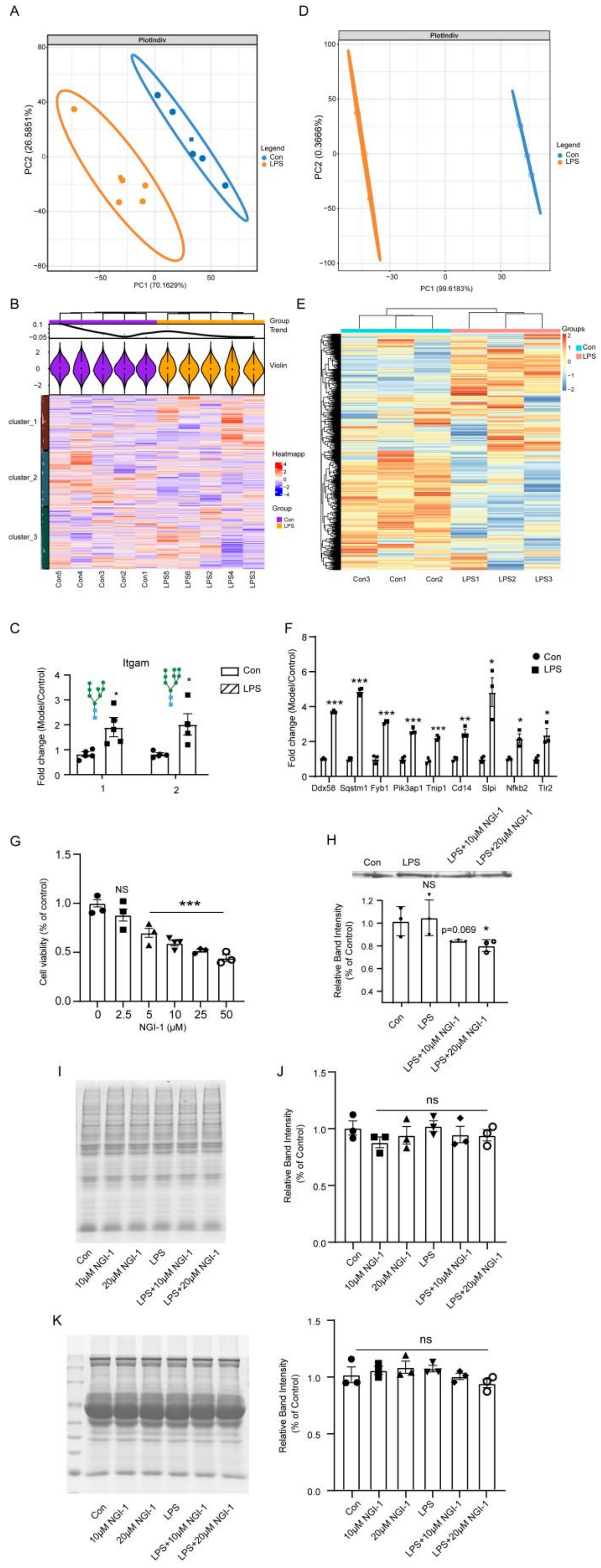

(A) PLS-DA score plot of proteins identified in brain tissue of LPS-treated and control group. (B) Heatmap analysis of hierarchical clustering of differentially expressed genes in two groups. Each row stands for one gene, and each column represents a time point. The Z-score method was used to

normalize the expressions of differentially expressed genes. The color and number (from -4 to 4) indicate the relative abundance of gene FPKM after normalization. Each group contained 5 samples. (C) N-glycan expression levels of Itgam changed. (D) PLS-DA score plot of proteins identified in BV2 cell of LPS-treated and control group. (E) Heatmap analysis of hierarchical clustering of differentially expressed genes in two groups. Each row stands for one gene, and each column represents a time point. The Z-score method was used to normalize the expressions of differentially expressed genes. The color and number (from -2 to 2) indicate the relative abundance of gene FPKM after normalization. Each group contained 3 samples. (F) Protein expression levels of Ddx58, Sqstm1, Fyb1, Pik3ap1, Tnip1, Cd14, Slpi, Nfkb2, and Tlr2 changed in each group. (G) Effect of a series of doses (2.5, 5.0, 10, 25 and 50 μM) of NGI-1 on cell viability for 24 h. Each group contained 3 samples. Error bars represent mean ± standard error (SE). Statistical analysis was performed by one-way ANOVA. P values indicating significance are depicted as follows: *$P < 0.05$, **$P < 0.01$, ***$P < 0.001$ *vs* 0 group. (H) Statistics of glycoproteins expression by ImageJ. (I-J) Expression levels of total proteins in NGI-1-treated and untreated cells with equal loading amounts. (K) Total protein expression in the secretome of BV2 cells. After incubating cells for 24 hours, collect the culture supernatant (containing FBS). Centrifuge and freeze-dry the supernatant to concentrate it. Determine the protein concentration using the BCA assay (after subtracting the effect of the blank medium). Standardize the loading volume and perform SDS-PAGE electrophoresis. Each group contained 3 samples. Error bars represent mean ± standard error (SE). Statistical analysis was performed by one-way ANOVA. P values indicating significance are depicted as follows: *$P < 0.05$, **$P < 0.01$, ***$P < 0.001$, ns is non-significant *vs* Con group.

Figure S2 Tanshinone IIA suppresses neuroinflammatory responses in both in *vivo* (mice) models and in *vitro* models.

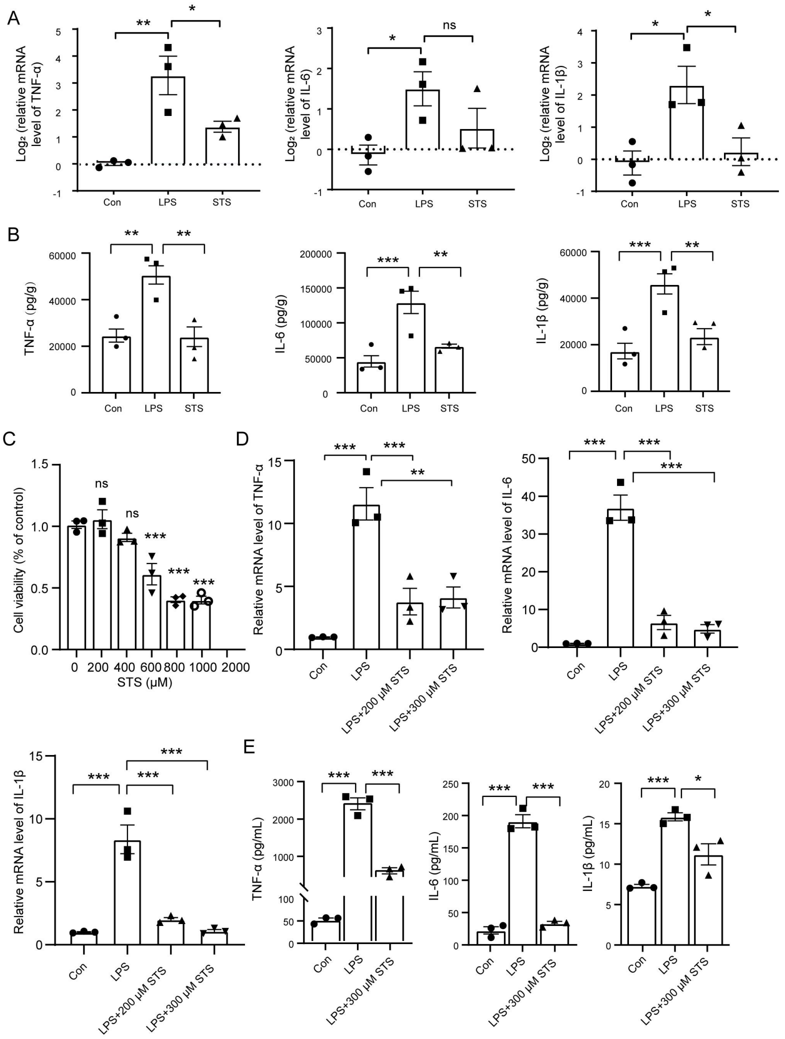

(A) TNF-α, IL-6 and IL-1β mRNA expression in mice brain was detected by qRT-PCR. Each group contained 3 samples. Three replicate wells were set up per sample. (B) ELISA results showed the protein expressions of TNF-α, IL-6 and IL-1β in mice brains. (C) Effect of a series of doses (0.2, 0.4, 0.6, 0.8, 1.0 and 2.0 mM) of STS on cell viability for 24 h. (D) The mRNA expression levels of TNF-α, IL-6 and IL-1β mRNA were detected in BV2 cells after STS treatment. Each group contained 3 samples. Four replicate wells were set up per sample. (E) The protein expression levels of TNF-α, IL-6 and IL-1β in BV2 cells after STS treatment. Each group contained 3 samples. Error bars represent mean ± standard error (SE). Statistical analysis was performed by one-way ANOVA. P values indicating significance are depicted as follows: *P < 0.05, **P < 0.01, ***P < 0.001, ns is non-significant *vs* LPS group.

Figure S3 Proteomic analysis of BV2 cells treated with Tanshinone IIA.

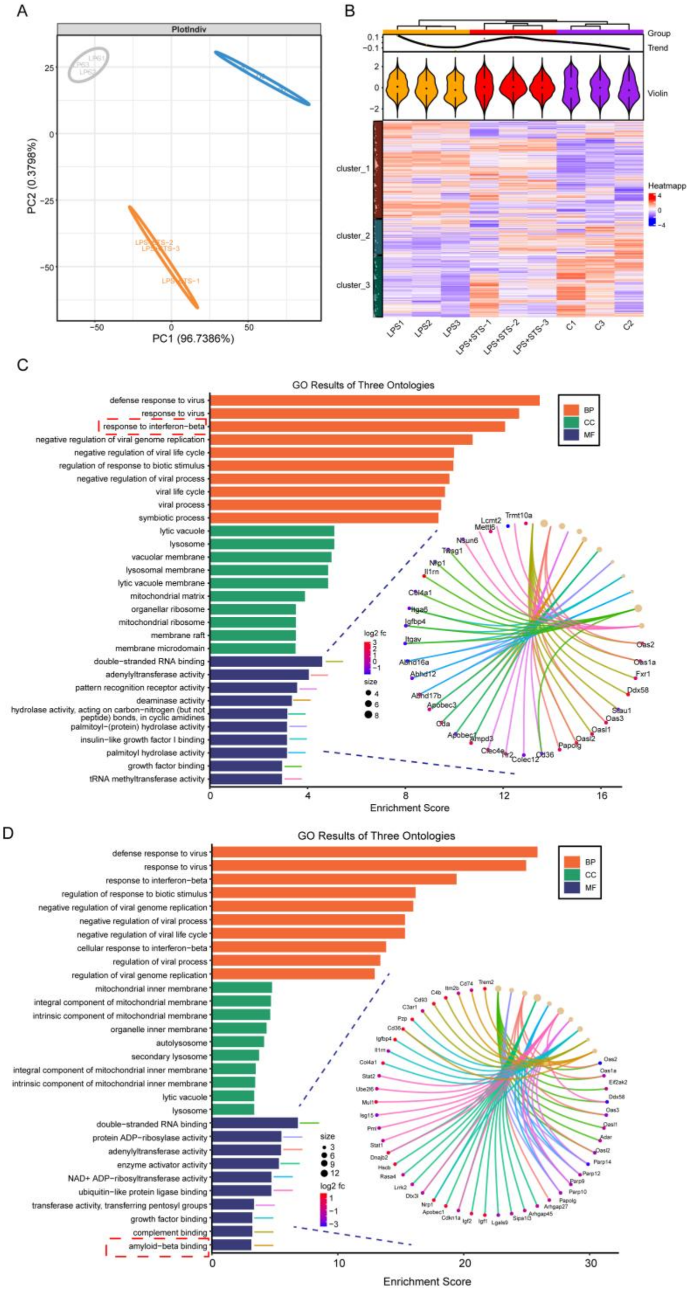

(A) PLS-DA score plot of proteins identified in brain tissue of LPS-treated, Tanshinone IIA-treated group and control group. (B) Heatmap analysis of hierarchical clustering of differentially expressed genes in three groups. Each row stands for one gene, and each column represents a time point. The Z-score method was used to normalize the expressions of differentially expressed genes. The color and number (from -4 to 4) indicate the relative abundance of gene FPKM after normalization. (C-D) GO enrichment analysis of BP, CC and MF in differentially expressed proteins in LPS group *vs* control group (C) or in LPS group *vs* Tanshinone IIA-treated group (D). Each group contained 3 samples.

Figure S4 The Effects of Tanshinone IIA on metabolites.

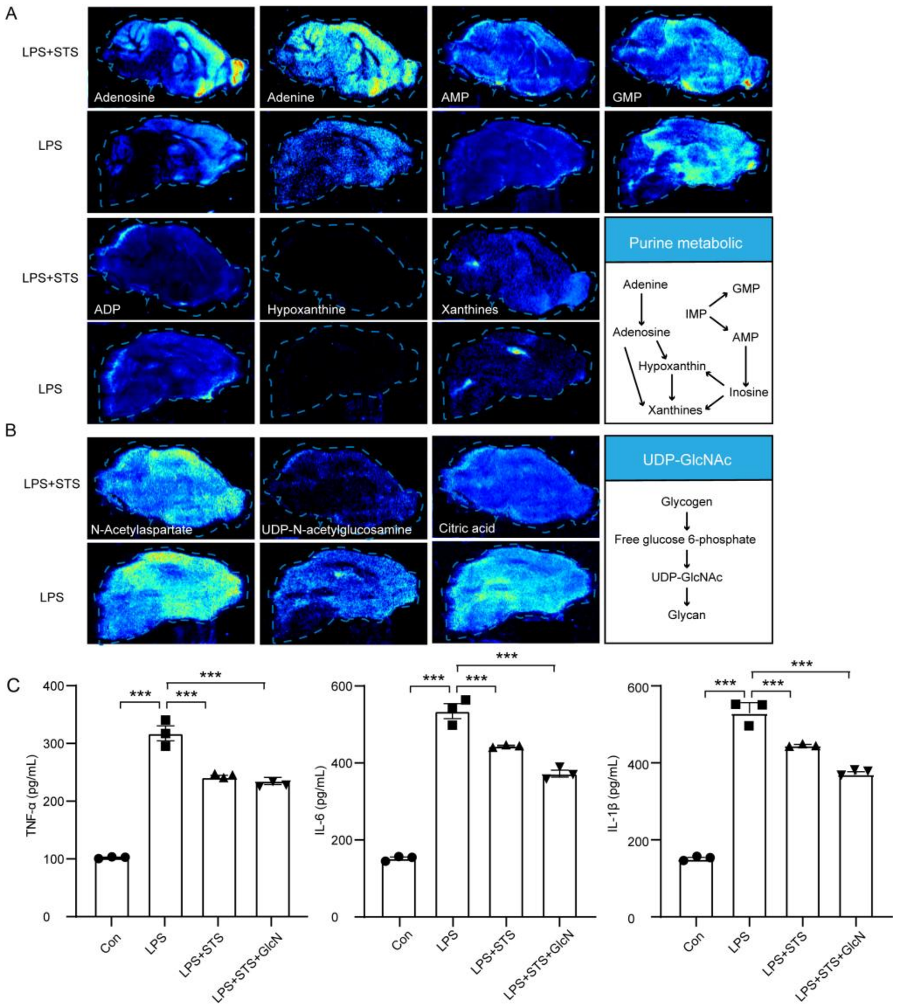

(A-B) Purine pathway and distribution of related metabolites in the mouse brain obtained by AFADESI-MSI. (C) The concentrations of TNF-α, IL-6 and IL-1β in the culture supernatants of primary microglia were determined by ELISA. Each group contained 3 samples. Error bars represent mean ± standard error (SE). Statistical analysis was performed by one-way ANOVA. P values indicating significance are depicted as follows: *P < 0.05, **P < 0.01, ***P < 0.001 *vs* LPS group. $^{\#}P$ < 0.05, $^{\#\#}P$ < 0.01, $^{\#\#\#}P$ < 0.001, ns is non-significant *vs* LPS+STS group.

Figure S5 Glycoproteome-wide profiling of Tanshinone IIA's effects on mouse brains.

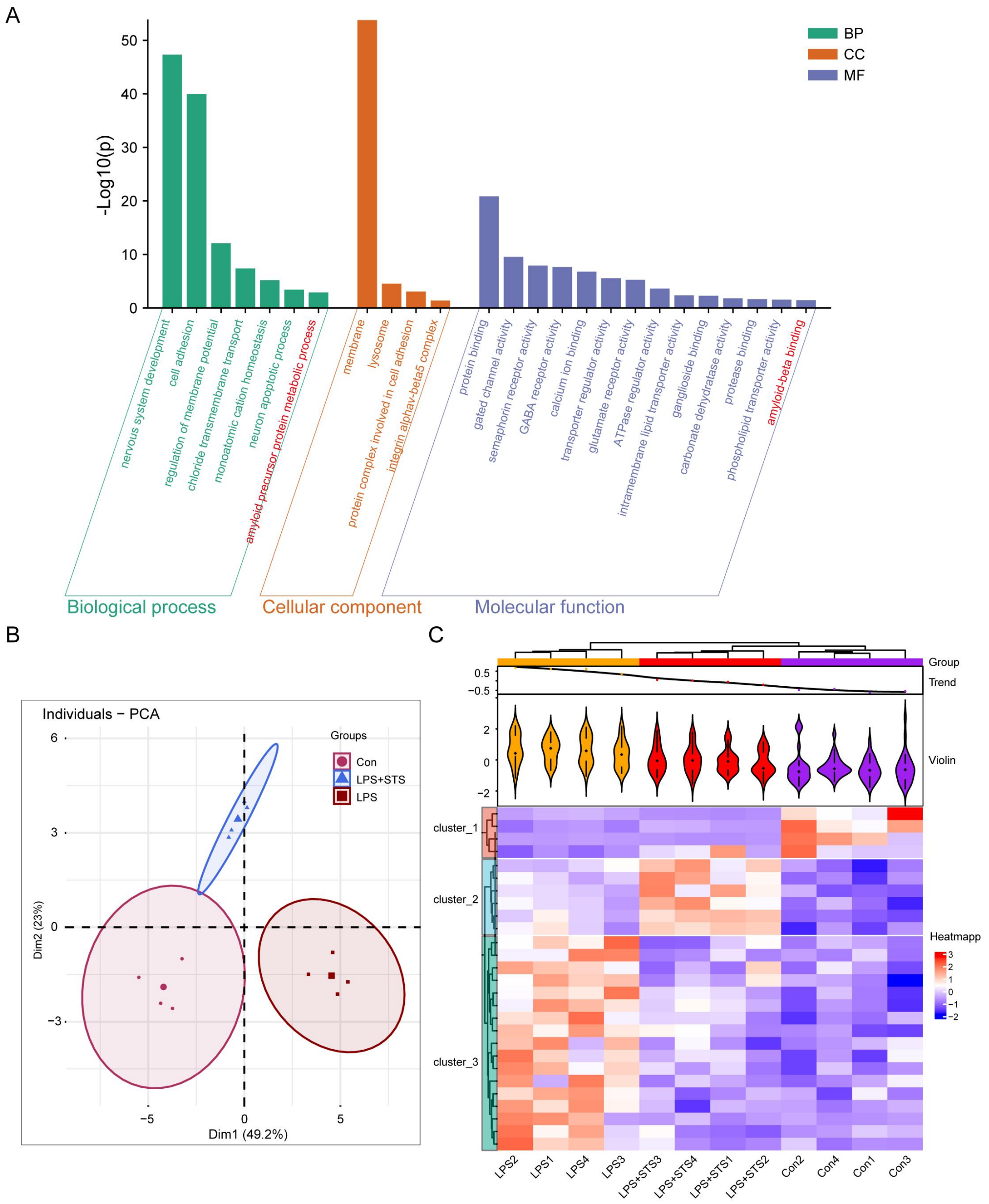

(A) GO enrichment analysis of glycoproteins corresponding to glycosylation site not recorded in the UniProt database. (B-C) PCA (B) and Heatmap (C) for the differentially expressed glycoproteins between Tanshinone IIA-treated, LPS group and control group in mice brain. Each group contained 4 samples.

Figuer S6 Tanshinone IIA inhibits neuroinflammation-induced alterations in glycoprotein levels.

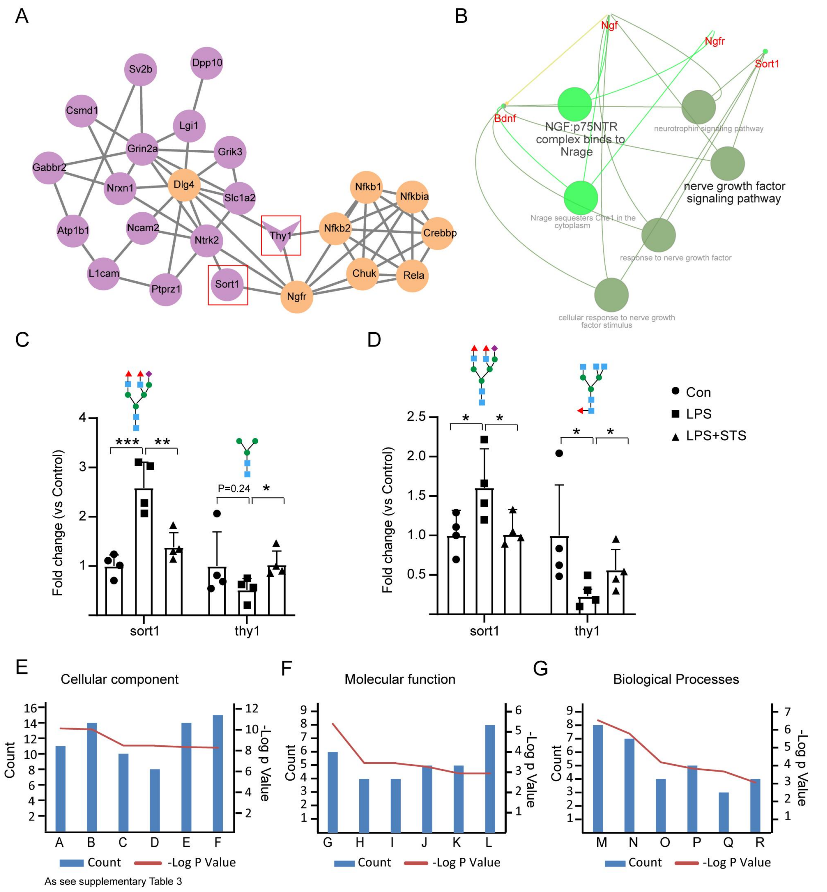

(A) NF-κB related PPI networks constructed. Each node represents a protein. Orange: added NF-κB related genes; purple: differential genes. (B) The enrichment pathways based on different glycoproteins were generated using ClueGO in cytoscape. Node colors represent the pathway terms and the size of the nodes indicates the significance of the term (larger means more significant). (C) N-glycan expression levels of sort1 and thy1 changed in each group. (D) O-glycan expression levels of sort1 and thy1 changed in each group. (E-G) Top six canonical pathways involving the dysregulated glycoproteins. (E) Cellular component. (F) Molecular function. (G) Biological processes. "Count" is the number of genes in the user-provided lists with membership in the given ontology term. "Log10(P)" is the p-value in log base 10. The glycan symbols are as follows: green circle for Hex, blue square for HexNAc, purple diamond for sialic acids and red triangle for fucose. Each group contained 4 samples. Error bars represent mean ± standard error (SE). Statistical analysis was performed by one-way ANOVA. P values indicating significance are depicted as follows: $*P < 0.05$, $**P < 0.01$, $***P < 0.001$ *vs* LPS group.

Table S1 Percentage of glycan content attached to glycosylation sites

Group	1 glycan accounted	2 glycan accounted	≥3 glycan accounted
Con	52.2%	19.4%	28.4%
LPS	46.6%	20.1%	33.3%

Table S2 Percentage of glycan content attached to glycosylation sites

Group	1 glycan accounted	2 glycan accounted	≥3 glycan accounted
LPS	46.6%	20.1%	33.3%
STS	48.7%	18.4%	32.9%

Table S3 GO functional enrichment analysis of differentially expressed mouse brain glycoproteins

Number	Name	*P* value	q value	FDR
A	glutamatergic synapse	1.10E-10	1.57E-08	1.18E-08
B	neuron projection	1.35E-10	1.57E-08	1.18E-08
C	axon	5.08E-09	3.11E-07	2.34E-07
D	axon part	5.34E-09	3.11E-07	2.34E-07
E	neuron part	6.98E-09	3.13E-07	2.35E-07
F	cell projection	8.05E-09	3.13E-07	2.35E-07
G	cell adhesion molecule binding	3.98E-06	8.76E-04	7.96E-04
H	sodium ion transmembrane transporter activity	3.48E-04	0.026471438	0.024064944
I	integrin binding	3.61E-04	0.026471438	0.024064944
J	metal ion transmembrane transporter activity	5.32E-04	0.029286855	0.026624414
K	inorganic cation transmembrane transporter activity	0.001130317	0.043404963	0.039459057
L	receptor binding	0.001183772	0.043404963	0.039459057
M	modulation of chemical synaptic transmission	6.37E-07	8.33E-04	7.78E-04
N	regulation of transmembrane transport	4.55E-06	0.002975545	0.002779755
O	regulation of neurotransmitter levels	1.27E-05	0.003193866	0.002983711
P	cell junction organization	1.36E-05	0.003193866	0.002983711
Q	calcium-mediated signaling	1.36E-05	0.003193866	0.002983711
R	endocytosis	1.47E-05	0.003193866	0.002983711

Table S4 Summary of Primers sequences at position 217

Gene	Primer	Sequence (5' - 3')
N217D	Forward	ACA TCG AAA CAC AAC GAT GAT ACC CAG CAC
	Reverse	GTG CTG GGT ATC ATC GTT GTG TTT CGA TGT
